# Co-Occurrence of Viruses, Plant Pathogens, and Symbionts in an Underexplored Hemipteran Clade

**DOI:** 10.3389/fcimb.2021.715998

**Published:** 2021-08-26

**Authors:** McKinlee M. Salazar, Mônica T. Pupo, Amanda M. V. Brown

**Affiliations:** ^1^Department of Biological Sciences, Texas Tech University, Lubbock, TX, United States; ^2^School of Pharmaceutical Sciences of Ribeirão Preto, University of São Paulo, Ribeirão Preto, Brazil

**Keywords:** membracid, treehopper, endosymbiont, plant pathogen, phage, metagenomics

## Abstract

Interactions between insect symbionts and plant pathogens are dynamic and complex, sometimes involving direct antagonism or synergy and sometimes involving ecological and evolutionary leaps, as insect symbionts transmit through plant tissues or plant pathogens transition to become insect symbionts. Hemipterans such as aphids, whiteflies, psyllids, leafhoppers, and planthoppers are well-studied plant pests that host diverse symbionts and vector plant pathogens. The related hemipteran treehoppers (family Membracidae) are less well-studied but offer a potentially new and diverse array of symbionts and plant pathogenic interactions through their distinct woody plant hosts and ecological interactions with diverse tending hymenopteran taxa. To explore membracid symbiont–pathogen diversity and co-occurrence, this study performed shotgun metagenomic sequencing on 20 samples (16 species) of treehopper, and characterized putative symbionts and pathogens using a combination of rapid blast database searches and phylogenetic analysis of assembled scaffolds and correlation analysis. Among the 8.7 billion base pairs of scaffolds assembled were matches to 9 potential plant pathogens, 12 potential primary and secondary insect endosymbionts, numerous bacteriophages, and other viruses, entomopathogens, and fungi. Notable discoveries include a divergent *Brenneria* plant pathogen-like organism, several bee-like *Bombella* and *Asaia* strains, novel strains of *Arsenophonus*-like and *Sodalis*-like symbionts, *Ralstonia* sp. and *Ralstonia*-type phages, *Serratia* sp., and APSE-type phages and bracoviruses. There were several short *Phytoplasma* and *Spiroplasma* matches, but there was no indication of plant viruses in these data. Clusters of positively correlated microbes such as yeast-like symbionts and *Ralstonia*, viruses and *Serratia*, and APSE phage with parasitoid-type bracoviruses suggest directions for future analyses. Together, results indicate membracids offer a rich palette for future study of symbiont–plant pathogen interactions.

## Introduction

Many insects that host microbial symbionts can also vector significant plant pathogens (Hogenhout et al., 2008a; Hogenhout et al., 2008b; Scholthof et al., 2011; Mansfield et al., 2012; Weintraub et al., 2019; Shafiq et al., 2020), but investigating the co-occurrence and forces underlying insect symbiont and plant pathogen infection presents a challenge. Some data suggest there could be an energetic cost of hosting symbionts, which might reduce an insect’s plant pathogen vectoring ability, while conversely, the benefits of symbionts might provide energy or metabolic advantages that increase plant pathogen transmission (Heck, 2018; Gonella et al., 2019). In developing a theory to predict the outcomes of these interactions, part of the challenge is their complexity. Insect symbioses tend to be richly multipartite, including many species of bacteria and fungi along with their phages and viruses. This complexity involves many insect body sites (gut, salivary glands, fat body, hemolymph, bacteriomes) and external ecological interactions that mediate symbiont and pathogen exchange. As a final surprising dimension of complexity in the dynamics of insect symbionts and plant pathogens, several studies show that insect symbionts can transfer through plant tissues and plant pathogens can evolve readily into insect symbionts (Chrostek et al., 2017; Martinson et al., 2020).

Hemipterans—especially aphids, whiteflies, psyllids, leafhoppers (Cicadellidae), and planthoppers (Fulgoromorpha)—are the most well-studied vectors of plant pathogens, but few studies have investigated treehoppers (Membracidae). Whereas related Auchenorrhyncha hemipterans typically host two bacteria in their symbiont organs (bacteriomes) that cooperatively synthesize missing essential amino acids not present in their plant-sap diet (Wu et al., 2006; Bennett and Moran, 2013; Douglas, 2016; Mao et al., 2017), there are exceptions. Some species have lost one of the obligate symbionts and gained a replacement symbiont (Sudakaran et al., 2017; Matsuura et al., 2018; Bell-Roberts et al., 2019). Other Hemiptera may have gained numerous symbionts. For example, light microscopy studies show Brazilian membracids host at least 28 diverse symbiotic microbes with up to six microbial species cohabiting a bacteriome (Rau, 1943; Müller, 1962; Buchner, 1965). It is unclear which of these microbes are primary or obligate from the host’s perspective, or secondary (facultative), functioning to enable their hosts to survive biotic or abiotic stresses or thrive in particular niches (Oliver et al., 2012; White et al., 2013; Oliver et al., 2014; Guidolin et al., 2018; Santos-Garcia et al., 2018; Lemoine et al., 2020). Many membracids further depend on additional behavioral symbioses with ants, bees, and wasps (Delabie, 2001; Godoy et al., 2006; Ibarra-Isassi and Oliveira, 2018; Klimes et al., 2018; Canedo-Júnior et al., 2019; dos Santos et al., 2019), which feed on the membracids’ secreted honeydew which, in turn, contains additional symbiotic microbes (Leroy et al., 2011; Fischer et al., 2015; Calcagnile et al., 2019; Shamim et al., 2019). However, there are few molecular studies of membracid symbioses. To date, there is one genomic study of a dual-symbiosis in a membracid (Mao et al., 2017) and one 16S rRNA and microscopy-based study showing three or four interacting microbial symbionts in two membracids (Kobiałka et al., 2019). Both studies, however, focused on membracids from the temperate climates, whereas membracid taxonomy, ecology, and microbiota are richest in the neotropics (Buchner, 1965; Cryan et al., 2000; Cryan et al., 2004; Deitz and Wallace, 2012; Hu et al., 2019).

A few membracids in North America are considered significant crop pests—specifically members of tribe Ceresini, such as *Spissistilus*, *Ceresa*, and *Stictocephala* (alfalfa and buffalo treehoppers) which cause swelling on stems or cuts that can facilitate infections in soybean and alfalfa (Meisch and Randolph, 1965; Bailey, 1975); however, in the neotropics, an estimated 18 genera of treehoppers are found as crop pests (Godoy et al., 2006). One study suggests *Ceresa* in Argentina may vector ﻿the witches’ broom phytoplasma (ArAWB) (Grosso et al., 2014). Further studies suggest membracids vector significant plant viruses: *Micrutalis* (tribe Micrutalini) vectors the viral pseudo-curly top disease (TPCTV) of tomatoes (Mead, 1986; Briddon et al., 1996) and *Spissistilus* vectors a closely related DNA virus, ﻿Grapevine red blotch-associated virus (GRBaV) (Bahder et al., 2016). Membracids’ roles as vectors of plant pathogens are not as well-studied as those of aphids, psyllids, and leafhoppers, which are more commonly found as crop pests. However, their predominance on woody plants makes membracids prime candidates worth investigating as possible vectors for phytoplasmas specific to woody plants such as 16SrIII, 16SrX (apple proliferation), or ESFY (European stone fruit yellows) (Wilson and Weintraub, 2007). Some studies suggest hemipteran secondary symbionts may travel between insects by passage through plants (Chrostek et al., 2017; Li et al., 2018; Pons et al., 2019), including *Wolbachia*, *Rickettsia*, *Candidatus* Cardinium, *Serratia*, and *Symbiopectobacterium* (formerly BEV), but these plant–insect–microbe exchanges have not been studied in membracids.

The current study sought to characterize symbionts and potential plant pathogens in membracids, including neotropical species, examining co-occurrence and correlations in abundance as an initial survey of this underexamined group. Our approach used shotgun metagenomics of the membracid bacteriome and surrounding tissues and hemolymph, searched for matches to major bacterial plant pathogens (Mansfield et al., 2012) including phytoplasmas (Hogenhout et al., 2008b), and DNA plant viruses (Shafiq et al., 2020), and an array of bacteriophage (Duron, 2014; Rouïl et al., 2020). Results showed our set of membracids hosted at least nine types of potential plant pathogens, in addition to 12 primary and secondary endosymbionts, along with numerous bacteriophages, and other viruses and parasites. Although our study focused on bacteriomes and surrounding hemolymph, we detected traces of phytoplasmas and spiroplasmas with no indication of plant viruses and we detected significant correlation between subsets of microbes present. Furthermore, we identified divergent *Brenneria* plant pathogen-like organisms, potential transitional strains of *Arsenophonus*-like symbionts, *Ralstonia* and *Ralstonia*-type phages, *Serratia*, and APSE-type phages and bracoviruses, indicating membracids may be host to a rich array of symbiont–plant pathogen interactions.

## Materials and Methods

### Insect Collection and Bacteriome Dissection

To obtain a phylogenetically diverse collection of membracids, sampling was performed over several years in the US and Brazil (permits and registration: SISBIO 46555-6; SisGen A350676 and R848FAD). Adult insects were collected by sweep net or by inspection of branches with capture in large plastic zip-closure bags. Sample collection site details are shown in **Supplementary Table S1**. Insects were preserved at -80°C prior to dissection in Brazil or the U.S. Insects were photographed and identified morphologically to genus or species prior to dissection. Microdissection to extract bacteriomes was performed on insects, one at a time, on ice trays with forceps and micron pins washed with bleach and 70–95% ethanol between each insect. For dissections, insects were placed in 100 µl of sterile phosphate buffered saline. Under 20–60X magnification, the posterior portion of the abdomen (~last three segments) was removed with forceps and a dissection needle, then tissue containing the bacteriomes was removed with micron pins and placed in labeled tubes, pooling several individuals collected at a single site together, to increase DNA yield (number of pooled individuals per sample: BM11 = 6, BM13-1 = 8, BM13-2 = 4, BM4 = 8, BM43 = 5, BM44 = 8, BM50 = 3, BM51 = 2, BM53 = 4, BM56 = 8, BM59 = 5, BM65 = 1, BM69 = 4, Cer = 4, Ent = 2, Gar = 8, MemA = 10, MemE = 12, MemM =16, Pub = 3). Due to the small size of bacteriomes, dissection was performed conservatively, allowing the inclusion of small amounts of surrounding abdominal hemolymph and host tissues including fat body cells.

### DNA Extraction and Illumina Library Preparation and Sequencing

To isolate and sequence DNA from pooled bacteriome tissues, we used either the Qiagen DNeasy Blood & Tissue Kit (Valencia, CA) or the Qiagen AllPrep DNA/RNA/miRNA Kit (Valencia, CA) following the manufacturer’s directions. DNA quantity and quality were assessed on the Nanodrop spectrophotometer. Library preparation for samples ‘Cer’, ‘Ent’, ‘Gar’, and ‘Pub’ was performed as described previously (Brown et al., 2014). For all other samples, libraries were prepared as follows: approximately 0.2 to 1 µg of DNA was used with the QIAseq FX DNA Library Kit (Valencia, CA) following the manufacturer’s directions except with modified fragmentation times and AMPure bead concentrations optimized to target 450–550 bp inserts. Library quality and quantity was assessed on the Agilent 2200 TapeStation. Libraries were normalized and pooled before sequencing on Illumina HiSeq, with 150 PE cycles performed at Genewiz, Inc (NJ).

### Sequence Assembly

To assemble reads for analysis, reads were filtered and trimmed using Trimmomatic v.0.38 (Bolger et al., 2014) and overlaps in paired reads were identified and joined together using Pear v0.9.11 (Zhang et al., 2014). Filtered paired and merged reads were *de novo* assembled with metaSPAdes v.3.13.0 (Bankevich et al., 2012; Nurk et al., 2017) using error correction and kmers (-k 25,33,43,53,65,87,101,115). Assembly quality and basic statistics were evaluated using Quast v5.0.1 (Gurevich et al., 2013).

### Database Searches

To identify microbes within the samples and confirm insect taxonomic identification, assembled scaffolds were processed by a three-step blast pipeline, using custom scripts. Briefly, to increase the speed of large blastn searches, scaffolds were first subjected to blastn in BLAST+ v2.10.1 (Camacho et al., 2009) against small custom target databases: a database of cytochrome oxidase (COI) genes from hemipterans, a database of 16S rRNA sequences from select bacteria including a wide range of endosymbionts, a database of 18S rRNA genes from fungi including a wide range of yeast-like symbionts, a database of phytoplasma/mycoplasma genomes downloaded from GenBank (NCBI; National Center for Biotechnology Information), and a virus and phage genome database compiled from the widest possible range of viral and phage genomes from NCBI. Samtools (Li et al., 2009) faidx was then used to extract the blast hit regions matching each custom database. These hit regions were then subjected to a second blastn search against all sequences in the nt database, extracting taxonomic data with the resulting hits. These hits were then filtered for the top blast match per scaffold, using a simple grep, to extract separately the full-length scaffolds corresponding to the desired hits matching taxonomic groups of interest (e.g., ‘bacteria’, ‘fungi’, ‘viruses’, ‘bugs’, ‘eukaryotes’, and ‘mycoplasmas’). Finally, the full scaffolds extracted above were subjected to a final blastn against the nt database to confirm that each scaffold matched the organismal clade previously identified.

### Abundance and Correlation Analysis

To assess the relative abundance and correlation between symbionts, plant pathogens, and other microbes within the membracids, we performed several filtering and analysis steps. First, we selected only scaffolds with top blastn hit to bacteria, fungi, insects, or viruses, removing any contaminants with high blastn sequence identity to common human microbiota or viruses. Next, we selected only scaffolds with blast hit length >40 bp and evalue >0.03 and bitscore >52. We then removed hits below 83% hit identity to the target (except with virus hits, for which we set a lower threshold of >70%). We also removed hits <48 bp length, except for phytoplasma and viruses, which we kept at the >40 bp threshold. To estimate and normalize coverage, kmer coverage was converted to absolute coverage with the equation C = (C_K_ R)/(R-K+1), where C is total coverage, C_K_ is kmer coverage, K is the length of kmers, and R is read length. For hits to the same species within a sample, coverages were added (i.e., combining variants) to assess total abundance. Final absolute coverages for all relevant hits were normalized based on the host insect’s COI gene coverage. Abundance and presence/absence were calculated, normalizing for within-sample abundance after COI normalization, plotting results using the function ‘heatmap’ in R. Spearman rank correlation was calculated for a matrix including all blastn hits, organized by taxa. Spearman rho values and p-values were calculated and plotted using several R packages: ‘Hmisc’ v4.5-0 (Harrell Miscellaneous) program ‘rcorr’ which calculates a matrix of Spearman’s rho rank correlation coefficients for all pairs of columns for non-missing elements, using midranks for ties, specifying method = “spearman”, corrplot with order = “hclust”, hclust.method = “average”, and for plotting, using packages ‘tidyr’, ‘tibble’, ‘ggplots2’, ‘corrplot’. P-value were corrected for multiple testing using the Benjamini and Hochberg (1995) method (FDR) in R with ‘p.adjust’ “BH”. Matching hits were classified into predicted categories (primary or secondary symbionts, entomopathogens or viruses, or potential pathogenic or beneficial plant associated microbes) based on metadata with matching references in NCBI (e.g., host insect or plant source and keywords indicating its role) combined with literature surveys on matching species or strains. Because some matches, such as *Pantoea*, *Enterobacter*, and *Serratia* can be potentially insect-associated, but are most often studied and reported as plant-associated, for the purposes of this study, we classified these as putative or potential plant pathogens. Similarly, those matching strains with possible beneficial functions or more rarely, other functions, were classified for this study as “potentially beneficial” plant-associated bacteria. These classifications are by no means definitive, but instead serve as a tentative best assessment of potential functional class.

### Phylogenetic Analysis

To confirm the taxonomic identity and evolutionary place of the membracids sampled, we extracted partial cytochrome oxidase I (COI) sequences from our scaffolds and performed phylogenetic analyses with other membracid and outgroup sequences downloaded from GenBank. While the COI mitochondrial locus is not ideal for inferring deep phylogenetic relationships in the Membracidae, it is useful as an abundant marker likely to produce sufficient coverage for samples with lower sequencing depth and provides databases of additional species for comparison. Resolving deep relationships among major membracid clades was not the primary goal of this study. COI sequences were aligned with Mafft v1.0.4 (Katoh and Standley, 2013) within the Geneious Prime v2020.0.4 (Biomatters, Ltd) suite. Maximum likelihood phylogenetic analysis was performed using RAxML v4.0 (Stamatakis, 2014) with the GTR Gamma nucleotide model, with rate heterogeneity alpha estimated, and with rapid bootstrapping and search for the best-scoring ML tree (-f a -x 1) with 100 replicates. Bayesian inference phylogenetic analysis was also performed on the same alignment block using MrBayes v2.2.4 (Huelsenbeck and Ronquist, 2001; Ronquist et al., 2012) with substitution model GTR+G with 4 categories, and Markov chain Monte Carlo settings of: chain length 1,100,000, 4 heated chains, heated chain temp 0.2, subsampling frequency 200, Burn-in length 100,000, with random seed 31,569, and priors with unconstrained branch lengths GammaDir (1,0.1,1,1), checking for convergence with minESS >200. Phylogenies were displayed using FigTree v1.4.4 (http://tree.bio.ed.ac.uk/software/), and image annotations were added in Adobe Illustrator.

To confirm microbial (bacterial and fungal) sequence identities and relationships, phylogenetic analyses were performed on 16S and 18S/28S rRNA regions extracted from our scaffolds, combined with the top 250 to 500 blastn hits from GenBank. Alignments and phylogenetic analyses were performed as described above for the COI region.

## Results

### Membracid Microbiota Diversity and Abundance

**Figure 1:**
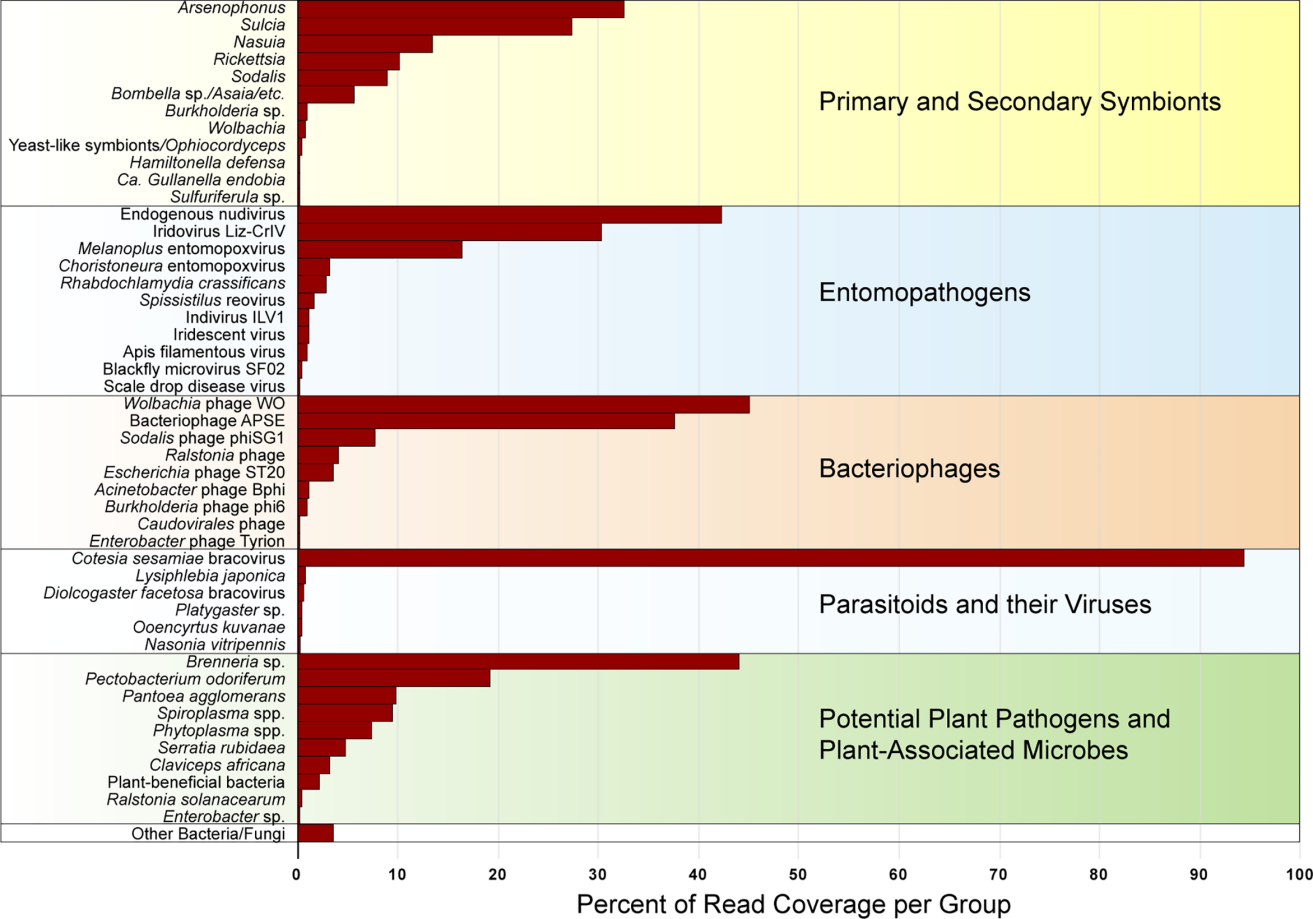
Relative abundance of primary and secondary membracid symbionts, entomopathogens, bacteriophages, parasitoids and their viruses, and potential plant pathogens (classified based on predominant function of closest blast hit), combined for all membracids, expressed as percentage of read coverage per each group shown in the figure, after normalizing between samples based on membracid cytochrome oxidase I (COI) gene coverage, calculated as a percentage of the read coverage for assembly scaffolds with blast hits to 16S rRNA genes (for bacteria), 18S rRNA genes (for fungi), or genomes for viruses and mycoplasmas or spiroplasmas.

In total, from the 20 sequenced treehopper samples comprising 8,609,965 scaffolds of >500 bp length adding to 8,708,441,348 bp of assembled sequence of which 5,539 scaffolds were over 10,000 bp and 189 scaffolds were over 50,000 bp with largest scaffold length 642,820 bp (see **Supplementary Table S2**), blastn top matches revealed over 133 potential strains or variants of bacteria, fungi, viruses, and parasites. There were 12 major groups of primary or secondary symbionts, with *Arsenophonus*, *Candidatus* Sulcia and *Candidatus* Nasuia (hereafter denoted simply *Sulcia* and *Nasuia*), *Rickettsia*, *Candidatus* Sodalis (hereafter denoted *Sodalis*), and *Bombella* sp. being the most abundant, based on 16S rRNA gene hits, in this order (**Figure 1**). Much less abundant symbionts included *Burkholderia*, *Wolbachia*, Yeast-like symbionts/*Ophiocordyceps*, *Candidatus* Hamiltonella (hereafter denoted *Hamiltonella*), *Candidatus* Gullanella (hereafter denoted *Gullanella*), and *Sulfuriferula* sp. There were 11 groups of potential entomopathogens including putative endogenous nudivirus, with the latter being most relatively abundant, based on viral genome hits, followed by Iridovirus Liz-CrlV, and entomopoxviruses (**Figure 1**). There were nine groups of bacteriophages, with *Wolbachia* phage WO and *Hamiltonella*-type APSE phages being most common, followed by *Sodalis* phage and *Ralstonia* phage (**Figure 1**). There were trace levels of parasitoid wasps, but relatively high levels of the *Cotesia*-type bracovirus (**Figure 1**). Among potential plant pathogens and plant-associated microbes, the most abundant groups were *Brenneria*-like and *Pectobacterium*-like strains, followed by *Pantoea agglomerans*, *Spiroplasma* spp., *Candidatus* Phytoplasma species (hereafter denoted *Phytoplasma* spp.), and *Serratia* sp. However, the *Spiroplasma* and *Phytoplasma* abundances were calculated based on genome-wide hits, because we did not find significant long 16S rRNA hits to either of these mycoplasma groups (**Figure 1**). Additional notable plant pathogens found include a distant match to the pathogenic fungi *Claviceps africana* and a highly similar match to the plant pathogen *Ralstonia solanacearum*.

### Relative Abundance of Potential Primary and Secondary Symbionts in Membracids

**Figure 2:**
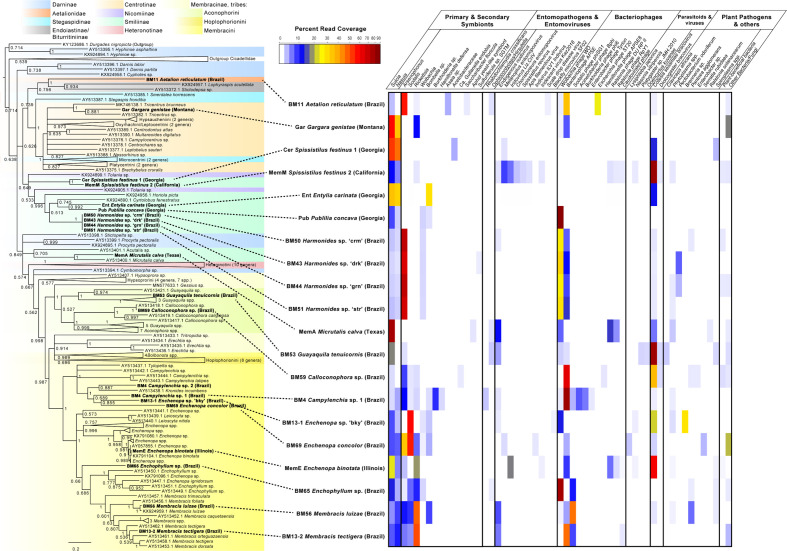
Phylogenetic tree and abundance plot of symbionts and plant pathogens of membracids in this study. Phylogeny is based on 956 aligned positions of the partial cytochrome oxidase I (COI) gene, generated using Bayesian 50% majority rule in MrBayes with GTR+G with 4 rate categories model, and showing posterior probabilities on branches (a similar topology with similar support was generated by RAxML GTR+Gamma with 100 bootstrap replicates). Specimens included in this study are shown in bold font. The presence and abundance plot is based on blastn hits to assembly scaffolds of symbionts, microbes, and pathogens, is depicted in the abundance heatmap as read coverage normalized as a percentage of the read coverage per scaffolds with blast hits from each sample, shown in color legend. Categories in abundance plot were classified based on predominant function of closest blast hit, and include putative primary and secondary membracid symbionts (bacterial and fungal); putative entomopathogens (including bacteria and fungi) and entomoviruses; bacteriophages, parasitoid wasps, and their viruses; potential fungal and bacterial plant pathogens; other bacteria and fungi.

Based on morphological identification, combined with COI blastn searches and phylogenetic analysis, our sampled treehoppers included 19 samples within family Membracidae, and one sample in a sister-family Aetalionidae, together comprising 8 tribes, 13 genera, and 16 species. The membracids sampled fell within subfamilies Centrotinae, Smiliinae, and Membracinae, in tribes Gargarini, Ceresini, Polyglyptini, Amastrini, Micrutalini, Aconophorini, and Membracini, most of which were supported clades in both Bayesian and Maximum Likelihood analyses (**Figure 2**). Presence/absence and abundance of microbes and pathogens within these samples shows a varied pattern of primary or secondary symbionts, entomopathogens, entomoviruses, bacteriophages, parasitoids and viruses, and possible plant pathogens (**Figure 2**). All species hosted the primary symbiont, *Sulcia*, with the average coverage of 282X, but with *Sulcia* occurring at varying abundances relative to other organisms (**Figure 2**). Phylogenetic analyses suggested these *Sulcia* symbionts formed a strongly supported monophyletic clade with similar phylogenetic topology to that of their hosts (**Supplementary Figure S1**). The second primary symbiont, betaproteobacteria *Nasuia*, appeared to be missing entirely for four species (*Aetalion reticulatum*, *Micrutalis calva*, *Guyaquila tenuicornis*, and *Calloconophora* sp.) (**Figure 2**). Remaining membracid *Nasuia* strains formed a well-supported monophyly with relationships that appear similar to those of the host (**Supplementary Figure S2**).

**Figure 3:**
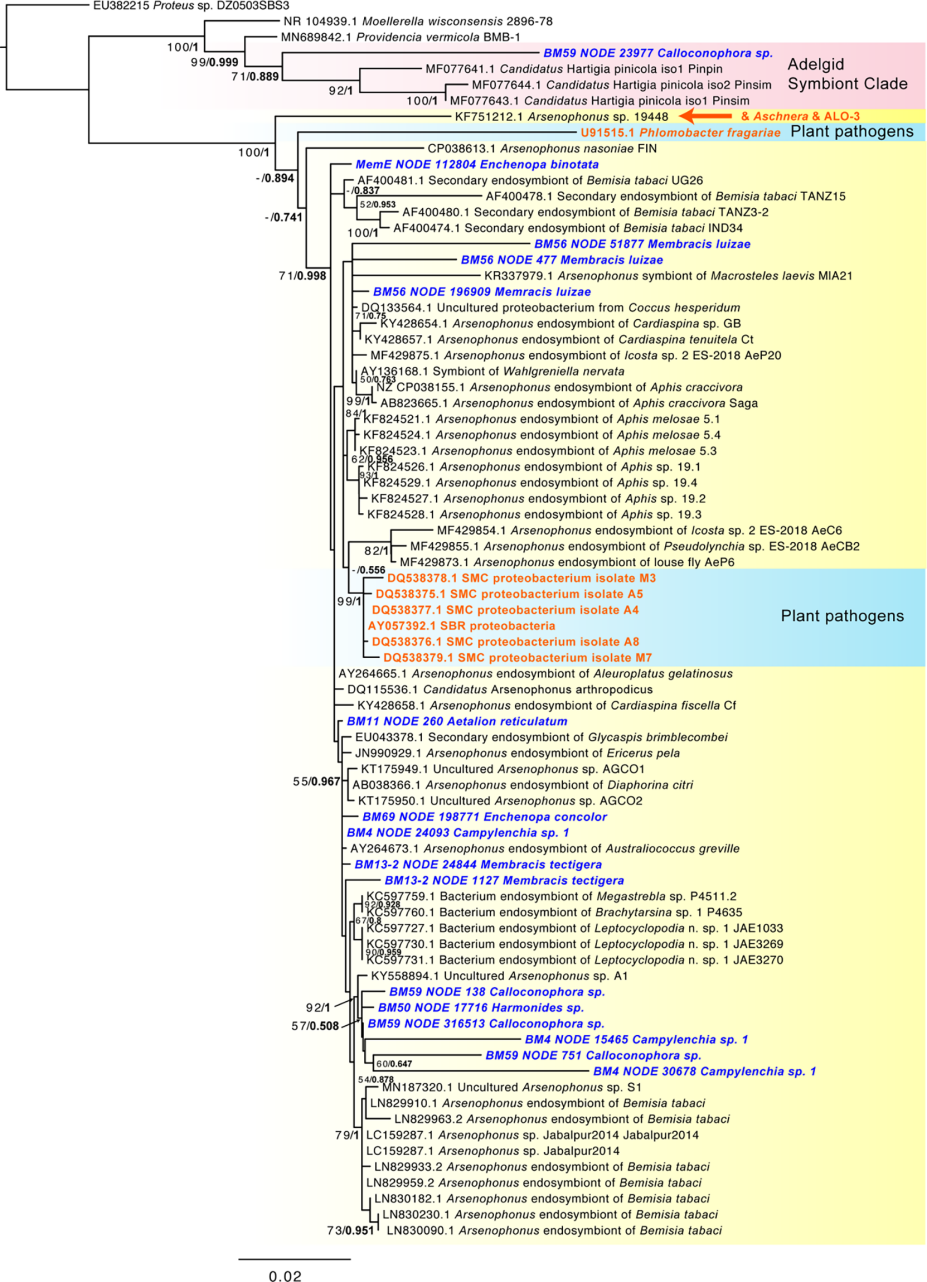
Phylogeny of *Arsenophonus*-like sequence based on 1,562 aligned positions of the 16S rRNA gene for sequences from GenBank and membracid samples in this study (bold blue font). Maximum likelihood phylogeny reconstruction was performed in RAxML GTR+Gamma with 100 bootstrap replicates. Supported nodes that were obtained from Bayesian 50% majority rule analysis in MrBayes with GTR+G with 4 rate categories are shown as values on branches as ML bootstrap/Bayesian posterior, with the latter values in bold font. Various *Arsenophonus*-like groups organisms are highlighted with color shading (yellow – most secondary symbiont *Arsenophonus*, red – clade with adelgid symbionts, blue – plant pathogenic organisms). The position of the obligate symbiont *Aschnera* and ALO-3 clade is indicated in orange font. A phylogeny including additional shorter scaffolds is shown in **Supplementary Figure S3**.

Common or abundant secondary or perhaps primary replacement symbionts included *Arsenophonus*, *Sodalis*, *Rickettsia*, *Wolbachia*, and *Bombella* sp. Rarer or occasional symbionts were *Burkholderia*, *Hamiltonella*, *Gullanella*, Acetobacteraceae/*Saccharibacter*, and *Sulfuriferula* sp. Phylogenetic analysis of *Arsenophonus* 16S rRNA (**Figure 3** and **Supplementary Figure S3**) showed most of these isolates clustered within a supported clade, with no monophyletic sub-clade for those strains from membracids; however, many samples contained more than one distinct sequence of the 16S rRNA gene. None of our sequences clustered with either of the two plant pathogenic groups of *Arsenophonus*-like organisms, *Candidatus* Phlomobacter fragariae and the ‘SMC proteobacterium isolates’, renamed *Ca.* Arsenophonus phytopathogenicus (**Figure 3**). Similarly, our sequences did not group with the *Aschnera* or ‘ALO-3’ clades comprising proposed obligate endosymbionts. One sample (BM59 *Calloconophora* sp.) included a more distantly placed *Arsenophonus*-like variant that clustered close to the adelgid endosymbiont clade *Candidatus* Hartigia pinicola. Fewer samples hosted *Sodalis*-like 16S rRNA sequences, but these formed no clear monophyly for strains from membracids, and several sequence variants were found within some samples (**Supplementary Figure S4**). *Rickettsia* formed two distinct clades: one clustered exclusively with hemipteran-host *Rickettsia*, and the other clade having polytomy with widely diverged insect hosts (**Supplementary Figure S5**). The *Wolbachia* sequences in these samples fell into supergroups A and B, widespread in insects (**Supplementary Figure S6**). Acetobacteraceae, including *Bombella* sp., *Asaia* sp., and *Saccharibacter* sp. were found in several samples at high abundances. Phylogenetic analyses of representative 16S rRNA gene sequences showed *Asaia* sp. in *Aetalion reticulatum* and a distinct clade of *Bombella* sp. in six membracid samples (**Supplementary Figure S7**). Five samples hosted yeast-like symbionts or potentially entomopathogenic fungi in the *Ophiocordyceps*-like groups, forming four distinct clusters, phylogenetically (**Supplementary Figures S8, S9**).

Closely related samples (i.e., in the same species or same genus) shown in **Figure 2** generally had more similar symbiont patterns, e.g., *Harmonides* sp., *Enchenopa* spp., and *Membracis* spp. This pattern was similar for bacteriophages, but not for entomopathogens and entomoviruses, except endogenous nudiviruses. Among bacteriophages, *Wolbachia* phage WO and the *Hamiltonella*-derived bacteriophage APSE were highly abundant in many samples, followed by *Sodalis* phage phiSG1. Parasitoid wasp-like reads were rare and at low abundance, but wasp bracoviruses (*Cotesia*-like and *Diolcogaster*-like) were common, occurring in all but four samples, and the *Cotesia*-like bracovirus was highly abundant in many samples.

### Presence of Potential Plant-Pathogenic Microbes and Viruses, and Plant-Beneficial Microbes

**Figure 4:**
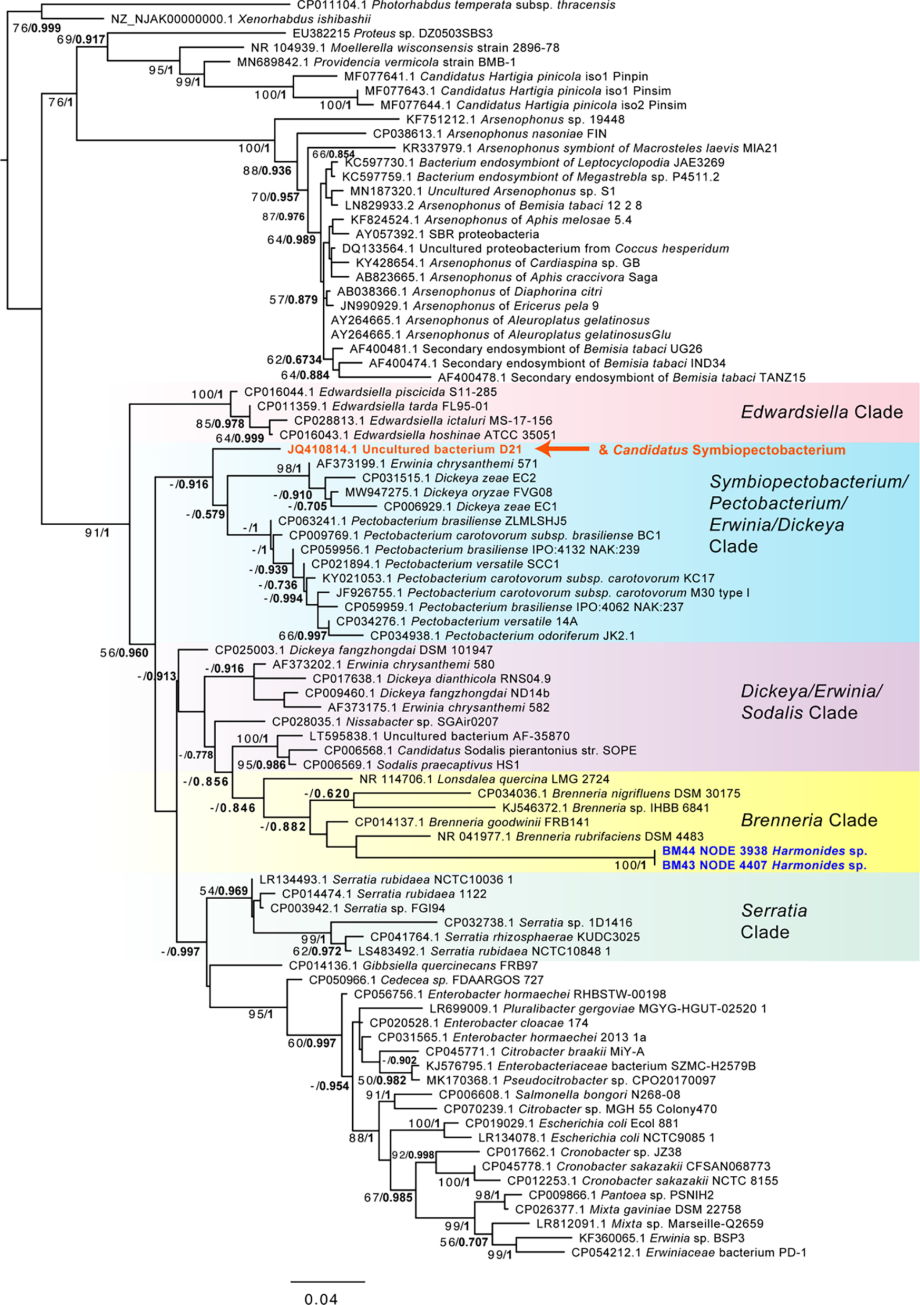
Phylogeny of Enterobacteriales including Pectobacteriaceae and *Brenneria*-like sequences based on 1,556 aligned positions of the 16S rRNA gene for sequences from GenBank and two membracid samples in this study with highest coverage (bold blue font). Maximum likelihood phylogeny reconstruction was performed in RAxML GTR+Gamma with 100 bootstrap replicates. Supported nodes that were obtained from Bayesian 50% majority rule analysis in MrBayes with GTR+G with 4 rate categories are shown as values on branches as ML bootstrap/Bayesian posterior, with the latter values in bold font. Various Pectobacteriaceae groups are highlighted with color gradient shading. The position of the *Candidatus* Symbiopectobacterium clade, comprising members with independent secondary endosymbiosis in insects and nematodes, is indicated in orange font.

Possible plant pathogens were identified at relatively low levels (**Figure 2**). One sample (*Guyaquila tenuicornis*) carried a fungal isolate distantly matched to the fungal plant pathogen *Claviceps africana*. Eleven samples carried one or more hits to *Phytoplasma* spp. or *Spiroplasma* spp. (**Table 1**). However, despite these scaffolds’ top blastn similarity to these mycoplasmas or spiroplasmas, many of the hits were short, and no 16S rRNA matches to either *Phytoplasma* spp. or *Spiroplasma* spp. were found. Several other putative plant pathogens were detected, based on 16S matches: *Pectobacterium* sp., *Brenneria* sp., *Pantoea agglomerans*, *Enterobacter* sp., *Serratia rubidaea*, and *Ralstonia solanacearum*. While many of the Pectobacteriaceae hits were low-coverage or consisted of short 16S rRNA matches, two samples (BM44 & BM43 *Harmonides* sp.) had high coverage scaffolds (>100X) that clustered consistently with *Brenneria* sp. in both maximum likelihood and Bayesian phylogenetic analysis (**Figure 4**), although with low bootstrap support and Bayesian posterior values. None of the Pectobacteriaceae hits in our samples clustered with the *Candidatus* Symbiopectobacterium group.

**Table 1:** *Phytoplasma* and *Spiroplasma* species and strains to which our membracid samples’ assembly scaffolds had top highest blastn matches, sorted in alphabetical order of strain names.

Phytoplasma or Sprioplasma strain	Sample name	Cov.	GenBank accession	E-value	Bit score	% Identity	Hit length	Scaffoldname	Scaffold length
*Ca. Phytoplasma australiense*	MemA	8.05	AM422018.1	0.005	56.5	86.8	53	NODE_136302	1433
*Ca. Phytoplasma mali* AT	BM13-1	4.04	CU469464.1	3.53E-04	58.4	90.9	44	NODE_909647	424
*Ca. Phytoplasma mali* AT	BM13-1	3.97	CU469464.1	1.04E-04	60.2	92.9	42	NODE_818945	446
*Ca. Phytoplasma mali* AT	BM13-1	4.95	CU469464.1	0.028	54.7	90.2	41	NODE_9454	2406
*Ca. Phytoplasma mali* AT	MemE	6.28	CU469464.1	0.007	56.5	89.1	46	NODE_106190	2145
*Ca. Phytoplasma mali* AT	MemM	4.91	CU469464.1	5.66E-17	100	89.9	79	NODE_2525275	416
*Ca. Phytoplasma ziziphi* Jwb-nky	BM4	2.82	CP025121.1	0.001	56.5	89.1	46	NODE_572066	413
*Ca. Phytoplasma ziziphi* Jwb-nky	BM51	2.16	CP025121.1	0.025	52.8	90.2	41	NODE_50151	622
*Ca. Phytoplasma ziziphi* Jwb-nky	BM56	11.25	CP025121.1	0.007	56.5	92.5	40	NODE_41289	2143
*Ca. Phytoplasma ziziphi* Jwb-nky	MemA	6.68	CP025121.1	0.023	54.7	90.5	42	NODE_65599	2038
Maize bushy stunt phytoplasma M3	BM44	2.91	CP015149.1	3.74E-04	58.4	92.7	41	NODE_256331	447
Onion yellows phytoplasma OY-M	MemM	4.87	AP006628.2	0.013	54.7	83.1	65	NODE_735674	1159
*Parthenium hysterophorus* phyllody phytoplasma PR08	MemE	7.30	CP060385.1	2.94E-04	60.2	91.1	45	NODE_238898	1202
*Parthenium hysterophorus* phyllody phytoplasma PR08	MemM	4.00	CP060385.1	4.06E-04	60.2	92.9	42	NODE_429229	1647
*Spiroplasma citri* strain C189	BM65	6.63	CP047426.1	0.002	58.4	86.2	58	NODE_13324	2620
*Spiroplasma citri* strain C5	BM53	5.64	CP053304.1	0.021	54.7	90.5	42	NODE_14244	1856
*Spiroplasma citri* strain C5	BM56	6.17	CP053304.1	6.38E-04	58.4	90.9	44	NODE_408898	738
*Spiroplasma citri* strain C5	BM56	9.68	CP053304.1	0.003	58.4	90.9	44	NODE_9283	3393
*Spiroplasma citri* strain C5	MemE	5.27	CP053304.1	1.26E-08	73.1	76.4	148	NODE_603041	423
*Spiroplasma citri* strain C5	MemE	5.23	CP053304.1	3.89E-05	62.1	82.3	79	NODE_467958	590
*Spiroplasma floricola* 23-6	Cer	4.20	CP025057.1	1.69E-11	82.4	76.8	155	NODE_33357	348
*Stolbur phytoplasma* 284/09	MemA	2.67	FO393427.1	0.002	56.5	92.5	40	NODE_341204	742

“Cov.” = scaffold read coverage. Hit and scaffold lengths are in base pairs.

Several of the bacteriophages detected in these data (**Figure 2**) appeared to be specific to these plant pathogens—particularly *Ralstonia* phages DU RP II and RpY1, and *Enterobacter* phage Tyrion. Many samples (12) were found to host other bacteria and fungi, based on 16S rRNA and 18S rRNA matches, for example, we found several hits matching typically plant-protective or plant-beneficial bacteria (e.g., in the groups *Proteus* spp., *Acinetobacter*, *Pseudomonas*, and Rhizobiales). Specific *Pseudomonas* matches were to strains naturally associated with potato, corn, and grass. In several samples, these unidentified (i.e., low blast similarity to named species) microbes were highly abundant. No plant viruses were found despite explicit searches against numerous DNA virus groups in our databases, including the Geminiviruses which are transmitted by a wide range of hemipterans and are often circulative in the insect.

### Correlations in Abundances of Possible Plant-Pathogens, Symbionts, Other Organisms, and Viruses

**Figure 5:**
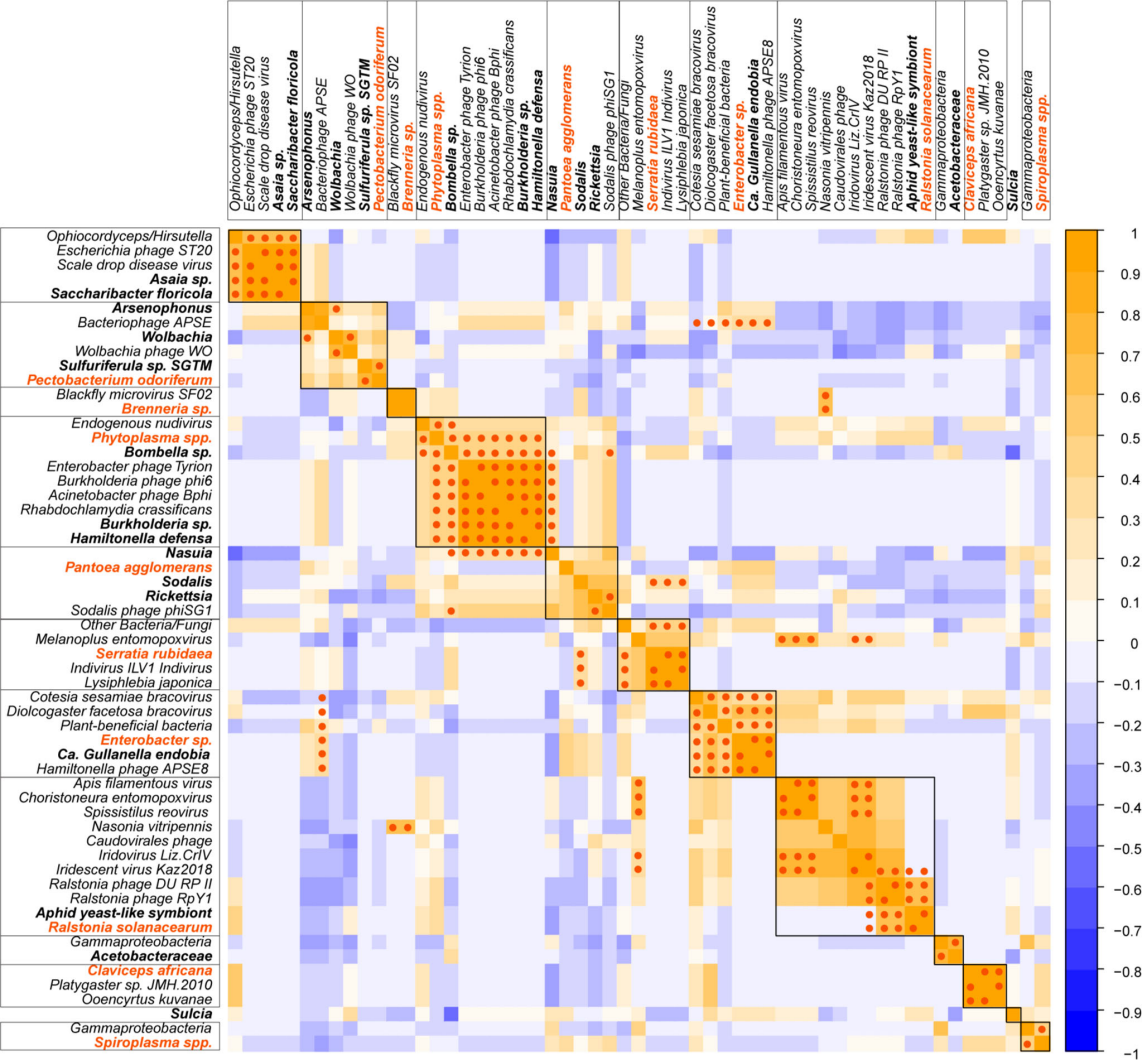
Spearman correlation heatmap of symbionts, plant pathogens, and other microbes and viruses in membracids. Abundances were normalized to the membracid’s cytochrome oxidase (COI) gene coverage before analysis of correlation. Spearman’s rho R-values are depicted with blue to orange shading, from negative to positive. Statistically significant p-values after FDR correction using the Benjamini and Hochberg (1995) method, showing values <0.05 are represented with dark orange dots. Putative plant pathogen names are depicted in dark orange bold font, and putative primary or secondary membracid symbiont names are depicted in black font.

Correlation analyses showed a range of positive and negative Spearman rho values for relative abundances of symbionts, plant pathogens, and other organisms and viruses within our membracids (**Figure 5**). The correlation plot of the rho values shows clusters of positively associated taxa (boxes in **Figure 5**), with larger clusters having putative plant pathogen taxa (dark orange font in **Figure 5**) for the *Pectobacterium* sp. + *Wolbachia/Arsenophonus*, *Phytoplasma* spp. +*Bombella/Burholderia/Hamiltonella*, *Enterobacter* sp. + *Gullanella*, and *Ralstonia* sp. + Aphid yeast-like symbiont. Within these groups, numerous entomopathogens and entomovirus taxa and phage were clustered.

Statistical support for Spearman rho, after Benjamini and Hochberg (1995) correction, produced several strongly positively associated taxa (high R-values) with p-values < 0.05 (**Tables 2**, **3**). Among correlations with putative plant-pathogenic taxa (**Table 2**), *Brenneria* sp. was significantly correlated with insect microvirus and a parasitoid, *Enterobacter* sp. was significantly associated with *Gullanella* and various phage and bracoviruses, *Ralstonia* sp. was associated with Aphid yeast-like symbionts and *Ralstonia*-type phages, *Serratia* sp. was associated with Indivirus and parasitoids, and *Phytoplasma* spp. was associated with *Hamiltonella*, *Burkholderia*, and several phages.

**Table 2:** Statistically significant correlations between relative abundances of putative plant-pathogens (classified based on predominant function of closest blast hit) and other organisms and viruses in membracids.

Possible plant pathogen	Correlated with	R-values	p-values
*Brenneria* sp.	Blackfly microvirus SF02	1	0
*Enterobacter* sp.	*Ca. Gullanella endobia*	1	0
*Ralstonia solanacearum*	Aphid yeast-like symbiont	1	0
*Enterobacter* sp.	*Hamiltonella* phage APSE8	1	0
*Serratia rubidaea*	Indivirus ILV1 Indivirus	1	0
*Serratia rubidaea*	*Lysiphlebia japonica*	1	0
*Enterobacter* sp.	Plant-beneficial bacteria	0.999775138	0
*Enterobacter* sp.	*Diolcogaster facetosa* bracovirus	0.99966794	0
*Enterobacter* sp.	*Cotesia sesamiae* bracovirus	0.99586849	0
*Ralstonia solanacearum*	*Ralstonia* phage RpY1	0.98284616	3.47E-13
*Ralstonia solanacearum*	*Ralstonia* phage DU RP II	0.958970237	7.30E-10
*Enterobacter* sp.	Bacteriophage APSE	0.923562133	1.46E-07
*Pectobacterium odoriferum*	Sulfuriferula sp. SGTM	0.873963533	1.02E-05
*Brenneria* sp.	*Nasonia vitripennis*	0.863438078	1.97E-05
*Serratia rubidaea*	Other Bacteria/Fungi	0.766561734	0.001457883
*Phytoplasma* spp.	*Bombella* sp.	0.725382699	0.004946463
*Phytoplasma* spp.	Acinetobacter phage Bphi	0.64893415	0.030102282
*Phytoplasma* spp.	Burkholderia phage phi6	0.64893415	0.030102282
*Phytoplasma* spp.	*Burkholderia* sp.	0.64893415	0.030102282
*Phytoplasma* spp.	*Hamiltonella defensa*	0.64893415	0.030102282
*Phytoplasma* spp.	*Rhabdochlamydia crassificans*	0.64893415	0.030102282
*Phytoplasma* spp.	Enterobacter phage Tyrion	0.64893415	0.030102282
*Phytoplasma* spp.	Endogenous nudivirus	0.640134401	0.035908217

Spearman’s ‘rho’ R-values and BH-FDR corrected p-values (<0.05).

**Table 3:** Statistically significant correlations between relative abundances of primary and secondary symbionts of membracids and other organisms and viruses in the samples.

Possible primary or secondary symbiont	Correlated with	R-values	p-values
Aphid yeast-like symbiont	*Ralstonia solanacearum*	1	0
*Asaia* sp.	*Escherichia* phage ST20	1	0
*Asaia* sp.	*Saccharibacter floricola*	1	0
*Asaia* sp.	Scale drop disease virus	1	0
*Burkholderia* sp.	*Acinetobacter* phage Bphi	1	0
*Burkholderia* sp.	*Burkholderia* phage phi6	1	0
*Burkholderia* sp.	*Enterobacter* phage Tyrion	1	0
*Burkholderia* sp.	*Enterobacter* phage Tyrion	1	0
*Burkholderia* sp.	*Hamiltonella defensa*	1	0
*Burkholderia* sp.	*Rhabdochlamydia crassificans*	1	0
*Ca. Gullanella endobia*	*Enterobacter* sp.	1	0
*Ca. Gullanella endobia*	*Hamiltonella* phage APSE8	1	0
*Hamiltonella defensa*	*Acinetobacter* phage Bphi	1	0
*Hamiltonella defensa*	*Burkholderia* phage phi6	1	0
*Hamiltonella defensa*	*Enterobacter* phage Tyrion	1	0
*Hamiltonella defensa*	*Rhabdochlamydia crassificans*	1	0
*Saccharibacter floricola*	*Escherichia* phage ST20	1	0
*Saccharibacter floricola*	Scale drop disease virus	1	0
*Ca. Gullanella endobia*	Plant-beneficial bacteria	0.999775138	0
*Ca. Gullanella endobia*	*Diolcogaster facetosa* bracovirus	0.99966794	0
*Ca. Gullanella endobia*	*Cotesia sesamiae* bracovirus	0.99586849	0
Aphid yeast-like symbiont	*Ralstonia* phage RpY1	0.98284616	3.47E-13
Aphid yeast-like symbiont	*Ralstonia* phage DU RP II	0.958970237	7.30E-10
*Asaia* sp.	*Ophiocordyceps/Hirsutella*	0.924240111	1.41E-07
*Saccharibacter floricola*	*Ophiocordyceps/Hirsutella*	0.924240111	1.41E-07
*Ca. Gullanella endobia*	Bacteriophage APSE	0.923562133	1.46E-07
*Wolbachia*	*Wolbachia* phage WO	0.888277599	3.69E-06
*Sulfuriferula* sp. SGTM	*Pectobacterium odoriferum*	0.873963533	1.02E-05
*Burkholderia* sp.	*Nasuia*	0.840735118	6.69E-05
*Hamiltonella defensa*	*Nasuia*	0.840735118	6.69E-05
*Nasuia*	*Acinetobacter* phage Bphi	0.840735118	6.69E-05
*Nasuia*	*Burkholderia* phage phi6	0.840735118	6.69E-05
*Nasuia*	*Enterobacter* phage Tyrion	0.840735118	6.69E-05
*Nasuia*	*Rhabdochlamydia crassificans*	0.840735118	6.69E-05
*Rickettsia*	*Sodalis* phage phiSG1	0.78471752	0.000791184
*Bombella* sp.	*Acinetobacter* phage Bphi	0.738623268	0.003379958
*Bombella* sp.	*Burkholderia* phage phi6	0.738623268	0.003379958
*Bombella* sp.	*Burkholderia* sp.	0.738623268	0.003379958
*Bombella* sp.	*Enterobacter* phage Tyrion	0.738623268	0.003379958
*Bombella* sp.	*Hamiltonella defensa*	0.738623268	0.003379958
*Bombella* sp.	*Rhabdochlamydia crassificans*	0.738623268	0.003379958
*Bombella* sp.	*Phytoplasma* spp.	0.725382699	0.004946463
*Bombella* sp.	*Endogenous nudivirus*	0.684129279	0.014570816
*Arsenophonus*	*Wolbachia*	0.676898466	0.017160695
*Bombella* sp.	*Sodalis* phage phiSG1	0.650415481	0.030102282
*Burkholderia* sp.	*Phytoplasma* spp.	0.64893415	0.030102282
*Hamiltonella defensa*	*Phytoplasma* spp.	0.64893415	0.030102282
*Bombella* sp.	*Nasuia*	0.633747909	0.040568676

Spearman’s ‘rho’ R-values and BH-FDR corrected p-values (<0.05). (several rows in this table are also shown in **Table 2**).

Among positively correlated primary and secondary symbionts and other taxa (**Table 3**), many of these associations were between bacterial symbionts and their presumptive phages. Other associations with symbionts were described previously, in **Table 2** and in clustered boxes described for **Figure 5**.

## Discussion

As a step toward discovering how symbionts impact vectoring of plant pathogens in an underexplored group of phloem-feeding insects, we performed this metagenomic sequencing study on membracids. We found, as expected based on historical studies of membracids from Brazil (Rau, 1943; Buchner, 1965), that these insects host a rich collection of primary and putative secondary symbionts, suggesting this group may be a model group for studying complex microbial interactions. Furthermore, from just 16 insect species using community metagenomics and a rapid blast pipeline, we found 12 potential symbiont clades, 9 groups of bacteriophages, 9 putative plant pathogen groups, and many other viruses and parasites, suggesting our approach could be promising if applied on a broader scale. We included membracid root taxa (*Aetalion*) (Cryan et al., 2000; Cryan et al., 2004; Costa, 2009; Dietrich et al., 2017; Evangelista et al., 2017; Skinner et al., 2020), taxa reported previously as symbiont-rich (*Enchophyllum*) (Rau, 1943), known virus-vectoring taxa (*Micrutalis*), and major crop pests (*Spissistilus* and *Ceresa*).

Putative plant pathogens included the ‘soft rot’ group of Enterobacteriales, *Brenneria* sp. and *Pectobacterium* sp., as well as other gammaproteobacteria such as *Enterobacter* sp., *Pantoea agglomerans*, and *Serratia* sp. Amongst these, *Brenneria* was most remarkable: *Brenneria* spp. are not previously known to be transmitted by insects, yet we found sequence matches that occurred at high coverage in two samples (*Harmonides* sp. membracids). Despite solid phylogenetic placement next to plant pathogenic *Brenneria* strains, the membracid *Brenneria*-like 16S rRNA gene sequences were highly divergent, suggesting an increased evolutionary rate, as is common in endosymbionts. However, without further study, we can only speculate on the features of this new *Brenneria* variant based on the group in which it is found. Importantly, *Brenneria* are relatives to three of the ‘top 10’ ranked plant pathogens *Erwinia*, *Dickeya*, and *Pectobacterium* (Mansfield et al., 2012) and are pathogens causing numerous diseases (cankers) of woody plants, including the deep bark canker of walnut (*Brenneria rubrifasciens*) and acute oak decline (*Brenneria goodwinii*) (Hauben et al., 1998; Bakhshi Ganje et al., 2021). They are noted for producing several unique compounds, such as the red pigment rubrifacine that may contribute to its virulence by inhibiting electron transport in mitochondria. *Brenneria* species also use sucrose to synthesize levan-type fructans for storage and defense (Liu et al., 2017), which may be of interest in hemipterans whose phloem diet is dominated by sucrose (Shaaban et al., 2020). The *Brenneria* strain did not, however, closely group with the broadly symbiotic group, *Symbiopectobacterium*, which includes recently evolved symbionts that independently colonized tissues of various arthropods and nematodes from plant pathogenic ancestors (Martinson et al., 2020; Vallino et al., 2021). Thus, the *Brenneria*-like sequences may reflect another independent case of a transition from plant pathogen to insect symbiont. Conversely, these sequences could simply be plant pathogens vectored by membracids.

Correlation analyses showed positive associations between the *Brenneria* and *Pectobacterium* strains and an insect microvirus, *Sulfuriferula*, and a parasitoid, but there was no other statistically supported association, suggesting no obvious interaction between primary or secondary symbionts and these Pectobacteriaceae. The *Serratia* strain found in this study was also correlated with a virus (Indivirus) and parasitoids. In contrast to the *Brenneria* strains, the *Serratia* strain showed high similarity (98% 16S rRNA) to *Serratia rubidaea*, a widespread plant pathogen. Notably, some *Serratia* species seem to circulate in both plants and hemipterans (Pons et al., 2019), for example, *Serratia symbiotica*, which likely helps its host digest plant proteins by secreting proteases (Skaljac et al., 2019). However, the *Serratia* strain occurred in one sample as a short scaffold and so any further analysis would require more data. *Pantoea*, a different enterobacterial plant pathogen that can be found in various settings including in insects (Walterson and Stavrinides, 2015), was found here only at very low coverage as a short ~85 bp match; therefore, it was not analyzed further. Similarly, while we found a sequence with 98.3% 16S identity to *Ralstonia solanacearum*, one of the top 10 plant pathogens, we found this in only a single sample (MemA *Micrutalis calva* from Texas). This *Ralstonia* was positively correlated with *Ralstonia*-type phages, as might be expected; however, Ralstonia-type phages were also found in two other U.S. samples (MemE *Enchenopa binotata* from Illinois and MemM *Spissistilus festinus* 2 from California), suggesting possible undetected *Ralstonia* in these samples. Phages of plant pathogens are increasingly becoming of interest for possible biocontrol of bacterial plant disease (Buttimer et al., 2017; Álvarez et al., 2019).

*Phytoplasma* species are wall-less phloem-infecting plant pathogenic bacteria that require both hemipteran insects and plants in their life cycles and occur in a wide range of woody plants; as such, they might be expected to occur in membracids. Their transmission and life cycle traits, and interactions with existing symbiont have been characterized in leafhoppers (Cicadellidae) and planthoppers (Hogenhout et al., 2008b; Ishii et al., 2013; Weintraub et al., 2019). Because Membracidae is a clade nested within the polyphyletic Cicadellidae (Skinner et al., 2020), authors have speculated that membracids might be expected to be important phytoplasma vectors (Wilson and Weintraub, 2007). Phytoplasmas appear most abundant in tropical and subtropical regions and one study from South America indicated a phytoplasma occurred in the membracid *Ceresa* (Grosso et al., 2014). Thus, it was surprising to find no 16S rRNA matches to *Phytoplasma* in these membracids, despite an initial search database of >5000 Phytoplasma 16S genes. Although we found eight samples with scaffolds having short matches to the large *Phytoplasma* genomic databases (including 1820 genomes), these are not strongly convincing that these membracids vector phytoplasmas. Although the path of these bacteria is through the stylet, intestine, hemolymph, and salivary glands (Weintraub et al., 2019), we expected that our bacteriome-focused dissections and sequencing depth would incidentally include phytoplasmas if they are present. With the caution that *Phytoplasma* vector status is ambiguous in these data, we note that there were some significantly positive associations in abundance between phytoplasmas and *Bombella* sp., phage, and other symbionts, suggesting perhaps ecologically common sources of these bacteria or bacterial fragments. Within order Hemiptera, *Spiroplasma* species reported thus far only from leafhoppers (Weintraub et al., 2019) where they can be either plant pathogens or be vertically transmitted as secondary symbionts, but we found short and low-similarity genome matches to this group as we did for phytoplasmas.

Whereas most viruses of plants have single-stranded RNA genomes and therefore would not be detected in this DNA sequence-based study, we searched for a wide range of DNA plant viruses, expecting to potentially discover some of these, especially those vectored by hemipterans including treehoppers (Mead, 1986; Briddon et al., 1996; Bahder et al., 2016; Varsani et al., 2017; Shafiq et al., 2020). We did not find any geminiviruses, including those related to *Topocurvirus* which includes the *Micrutalis*-vectored pseudo-curly top virus, TPCTV, or *Grablovirus* which includes the *Spissistilus*-vectored Grapevine red blotch-associated, GRBaV. Although most Geminiviridae are persistent or semipersistent and circulative in their hosts (Shafiq et al., 2020), therefore potentially found in the hemolymph or tissue surrounding the bacteriomes in this study, these are ssDNA viruses that form a dsDNA intermediate in the plant host but may not form a dsDNA phage in the insect, unless they are propagative. For most geminiviruses, it is not clear if they are propagative in the host. We also did not detect Caulimoviridae, which are dsDNA reverse transcribing viruses mostly transmitted by a range of hemipterans (Shafiq et al., 2020), although it is unclear how many of these viruses are circulating and propagative in the insects, suggesting perhaps these plant viruses could be vectored by these treehoppers but not easily detected by these methods.

Among the most abundant presumed secondary symbionts, we found these membracids to be dominated by the genera *Arsenophonus*, *Rickettsia*, *Sodalis*, and *Bombella*, each of which has members that can be found within plants or causing pathogenicity to plants (Crotti et al., 2016; Chrostek et al., 2017; Gonella et al., 2019). In general, secondary symbionts are diverse functionally, often enabling their hosts to survive a wide range of biotic or abiotic stresses (Gottlieb et al., 2008; Oliver et al., 2012; White et al., 2013; Oliver et al., 2014; Su et al., 2015; Sudakaran et al., 2015; Guidolin et al., 2018; Santos-Garcia et al., 2018; Lemoine et al., 2020). The prevalence, abundance, and phylogenetic analyses presented herein provide some hints, and many open questions, about how these bacteria function in these samples. The high abundance of *Arsenophonus* in these data has several possible explanations: its 16S rRNA gene occurs in multiple copies per genome (Sorfová et al., 2008) rather than as a single copy as for many endosymbionts including primary symbionts *Sulcia* and *Nasuia*, and remarkably *Arsenophonus* might occur as an abundant hypersymbiont living nested within the cells of the primary symbiont *Sulcia* (Kobiałka et al., 2016), in which case it could occur at high copy number. Regardless, the abundant and phylogenetically dispersed place of most of these *Arsenophonus*-like sequences suggest the pattern typical of facultative symbionts (Nováková et al., 2009). The lack of similarity to plant-pathogenic *Arsenophonus*-like organisms (*P. fragariae* and Ca. Arsenophonus phytopathogenicus, formerly ‘SMC proteobacteria’) suggests it is unlikely that these strains play this role. Similarly, the lack of similarity to the two probable obligate *Arsenophonus*-like organisms, *Aschnera* and ALO-3 (Duron, 2014; Santos-Garcia et al., 2018), suggests no evidence for this role in the sampled membracids. However, the discovery of one variant that clusters at the root of the adelgid symbiont clade (*Ca.* Hartigia pinicola) as sister to outgroup pathogens suggests a potentially distinct or perhaps parasite-to-commensal transitional function in this organism. Additionally, the typical *Arsenophonus* strains appear to have at the root of the tree a variant (accession KF751212.1) symbiotic in *Stomaphis* spp., which are hemipterans specializing on stems and roots of trees, raising the question of this diet as ancient in the *Arsenophonus* hosts. Ultimately, multi-locus *Arsenophonus* phylogenomics will be important in uncovering these relationships more accurately, particularly because the 16S rRNA tends to multi-copy in this group (Sorfová et al., 2008) along with comparative omics analysis and detailed microscopy to understand these symbionts, particularly given the observation that *Arsenophonus* can live within the cells of the primary symbiont *Sulcia* (Kobiałka et al., 2016).

Our finding of distinct strains of the symbiotic acetic acid bacteria (Acetobacteraceae).

*Bombella* (formerly *Candidatus* Parasaccharibacter apium)*, Asaia*, and *Saccharibacter floricola*, is a novel finding and of special interest in the sugar metabolism of these insects which secrete sugary honeydew to engage trophobiosis with ants and bees. *Saccharibacter floricola* (Jojima et al., 2004) specifically is bee-associated (Smith et al., 2020). *Asaia* and *Bombella* spp. occur in tropical plants and can be plant growth promoters (Crotti et al., 2016), whereas in insects they can stimulate the innate immune system, increase the rate of larval development, and provide insecticide resistance (Chouaia et al., 2010; Chouaia et al., 2012; Mitraka et al., 2013; Comandatore et al., 2021). They can transmit horizontally and vertically, crossing from the gut to the hemolymph and eggs. As symbionts, they likely play a major role in metabolizing sugars to acids (Dong and Zhang, 2006; Crotti et al., 2010); thus, we hypothesize this group to be potentially very important in the observed strong associations between ants and bees and the seven samples of membracids in which we found them. Based on the level of sequence divergence between the membracid *Bombella* isolates and other *Bombella* sp. together with the fact that most of the *Bombella* sequences were similar or identical, we speculate that there may be horizontal transfer of *Bombella* either between these membracids and their tending hymenopteran insects, or with plants.

Other symbionts exhibited patterns typical for their respective groups, as they occur in other Hemiptera. For example, *Rickettsia* was dispersed amongst samples and the phylogenetic tree as a typical facultative, partially vertically transferred symbiont, with similar sequences within a host-species. In most cases, *Rickettsia* is considered parasitic, but there is also evidence that some strains may confer survival benefits (Hendry et al., 2014). *Sodalis* showed a similar pattern in these data, with phylogenetically dispersed strains, consistent with multiple environmental to secondary symbiont transitions. However, two 16S rRNA gene sequences matching *Sodalis*, both from the membracid *Membracis tectigera* (BM13-2), displayed significant sequence divergence, which could suggest a transition in these strains from secondary to primary endosymbiont (Toju et al., 2010), or possibly pseudogenization of 16S rRNA gene copies. Although we found several matches to other symbionts (*Wolbachia*, *Burkholderia*, *Hamiltonella*, *Gullanella*, and *Sulfuriferula* sp.), the most notable of these was a high coverage 99.02% 16S rRNA match to a *Burkholderia* strain that is endophytic, living within the tissues of palm leaves. The strain, initially named *Burkholderia* sp. JS23, was re-named *Chitinasiproducens palmae* (Madhaiyan et al., 2020). Its occurrence at high coverage in this membracid sample is a mystery, but interestingly, this same Burkholderiaceae clade includes the *Mycoavidus* bacteria, which are endohyphal bacteria of the fungus *Mortierella elongata* (Ohshima et al., 2016).

As expected, all sampled membracids hosted *Sulcia*, which almost certainly serves to synthesize amino acids missing from the bugs’ phloem diet, but four samples were missing the betaproteobacteria co-symbiont *Nasuia*, that normally cooperatively synthesizes the remaining amino acids (Bennett and Moran, 2013; Douglas, 2016; Mao et al., 2017). Given the scope of the present study, it remains unclear whether other bacteria or yeast have become replacement symbionts in samples that are missing *Nasuia*. However, it is noteworthy that we discovered four distinct sequences matching *Ophiocordyceps/Hamiltonaphis*-like symbionts, three of which occurred in species that were missing *Nasuia* (BM11 *Aetalion reticulum*, MemA *Micrutalis calva*, and BM53 *Guyaquila tenuicornis*). Numerous studies from aphids, leafhoppers, and other hemipterans suggest that yeast-like symbionts are common and can emerge as co-symbionts or replacement symbionts (Fukatsu and Ishikawa, 1996; Suh et al., 2001; Sacchi et al., 2008; Nishino et al., 2016; Meseguer et al., 2017; Matsuura et al., 2018).

In addition to the *Ralstonia* phage discussed previously, two other groups of phages were highly abundant: the *Hamiltonella*-type APSE phages and *Wolbachia* phage WO, and in general, we found correlation in abundances between bacteria and their presumptive phages, as expected. The APSE phage, well-studied for its parasitoid-protective toxin in *Hamiltonella defensa* (Oliver et al., 2012; Oliver et al., 2014; Su et al., 2015), particularly in aphids and whiteflies (Rouïl et al., 2020), is commonly integrated into *Arsenophonus* through lateral gene transfer (Duron, 2014). The infrequency of *Hamiltonella* and widespread occurrence of APSE in these membracids suggest perhaps *Arsenophonus* serves as the APSE host in these species. Despite this potential protection from parasitoids conferred by the APSE phage, these membracids showed signs of parasitoid infection, particularly in the level of wasp-specific bracoviruses, especially *Cotesia*-type bracovirus. These dsDNA viruses act as mutualists for their wasp hosts, contributing to immune suppression of the parasitized insect once injected along with the wasp’s eggs. While bracoviruses are best-known from braconid wasps specifically parasitizing Lepidoptera, discoveries of these virus sequences in Hemiptera previously (Peng et al., 2011; Cheng et al., 2014) and in the present study raise interest in further study of these viruses and related Polydnaviridae in Hemiptera. The high bracovirus levels in these data could also arise from bracovirus horizontal gene transfers into the membracids, as has been observed in other hosts (Cheng et al., 2014; Chevignon et al., 2018). The low levels of parasitoid DNA compared with parasitoid virus DNA in these data could also be explained by our focus on bacteriome tissue, from which most parasitoid wasp DNA, if present, would likely be missed. Finally, despite the opposite biological effects of these two abundant viruses, APSE and bracoviruses, in our data, rather than being negatively correlated, they were significantly positively correlated.

In conclusion, genomic sequence analysis of this kind cannot directly predict insect vector capacity nor microbe pathogenicity; however, these genomic analyses can be invaluable for uncovering previously overlooked microbial associations. Although membracids have been long studied for their exceptional morphological traits, such as the elaborate pronotum, there is scant data on their microbial associations and vectoring potential. These results showed a rich array of microbes and viruses, including plant pathogens and potential allies, painting a preliminary picture of some critical taxa and interactions worth further research. Furthermore, this study generated a large amount of assembled genomic data with thousands scaffolds that are long enough for future in-depth analysis of gene content. We suggest future studies should investigate the prevalence, function, and mechanisms of these potentially interacting microbes, and potential vectored microbes identified here, such as the *Brenneria*-like bacteria, *Serratia*, *Ralstonia*, mycoplasmas or spiroplasmas, and various associated phages. Specifically, co-occurrence patterns leave uncertainty as to role and function of these microbes which should be addressed with phylogenomic analyses, FISH and TEM microscopy, comparative genomics and studies to assess possible HGTs and pseudogenes, and of course wherever possible also controlled infection experiments.

## Data Availability Statement

The datasets presented in this study can be found in online repositories. The names of the repository/repositories and accession number(s) can be found below: NCBI accession: PRJNA733715, SAMN19458266-SAMN19458285.

## Author Contributions

AB led the design of the study, developed bioinformatics code and pipelines, and drafted the manuscript. MP led the work and field collection in Brazil and assisted with student and laboratory infrastructure and specimen cataloging. MS led the library preparation on Brazilian specimens and assisted in writing the manuscript. All authors contributed to the article and approved the submitted version

## Conflict of Interest

The authors declare that the research was conducted in the absence of any commercial or financial relationships that could be construed as a potential conflict of interest.

## Publisher’s Note

All claims expressed in this article are solely those of the authors and do not necessarily represent those of their affiliated organizations, or those of the publisher, the editors and the reviewers. Any product that may be evaluated in this article, or claim that may be made by its manufacturer, is not guaranteed or endorsed by the publisher.
