# Supplementary material for: Co-Occurrence of Viruses, Plant Pathogens, and Symbionts in an Underexplored Hemipteran Clade
Source: Front Cell Infect Microbiol. 2021 Aug 26;11:715998. doi: 10.3389/fcimb.2021.715998 (PMC8426549; doi:10.3389/fcimb.2021.715998)

**SUPPLEMENTARY TABLES AND FIGURES:**

**Supplementary Table 1**: Insect collection details including sampled insects, habitats, and locations.

| **Sample Name** | **# Pooled** | **Habitat or Plant** | **Tending ants/bees** | **Location** | **Lat/Long** |
| --- | --- | --- | --- | --- | --- |
| BM11 | 6 | *Schinus terebinthifolius* | Bees/Ants | Ribeirão Preto, Brazil | -21.16006/ -47.863994 |
| BM13-1 | 8 | Mixed tropical shrubs | Ants | Ribeirão Preto, Brazil | -21.16006/ -47.863994 |
| BM13-2 | 4 | Mixed tropical trees | Bees/Ants Wasps/Flies | Ribeirão Preto, Brazil | -21.16006/ -47.863994 |
| BM4 | 8 | Various Mimosoideae | Ants | Ribeirão Preto, Brazil | -21.16006/ -47.863994 |
| BM43 | 5 | Mixed tropical shrubs | Ants | Ribeirão Preto, Brazil | -21.16006/ -47.863994 |
| BM44 | 8 | Mixed tropical shrubs | Ants | Ribeirão Preto, Brazil | -21.16006/ -47.863994 |
| BM50 | 3 | Mixed tropical shrubs | Ants | Ribeirão Preto, Brazil | -21.16006/ -47.863994 |
| BM51 | 2 | Mixed tropical shrubs | Ants | Ribeirão Preto, Brazil | -21.16006/ -47.863994 |
| BM53 | 4 | Mixed tropical shrubs | Bees/Ants Wasps | Ribeirão Preto, Brazil | -21.16006/ -47.863994 |
| BM56 | 8 | *Bauhinia variegata* | Bees | Ribeirão Preto, Brazil | -21.16006/ -47.863994 |
| BM59 | 5 | *Bauhinia variegata* | Bees | Ribeirão Preto, Brazil | -21.16006/ -47.863994 |
| BM65 | 1 | *Drypetes* sp. | none | Ribeirão Preto, Brazil | -21.16006/ -47.863994 |
| BM69 | 4 | Cyperoideae | Ants | Ribeirão Preto, Brazil | -21.16006/ -47.863994 |
| Cer | 4 | Roadside meadow | none | Near Dillard, GA, USA | 34.992874/ -83.382636 |
| Ent | 2 | Roadside shrubs | Ants | Atlanta, GA, USA | 33.787779/ -84.322729 |
| Gar | 8 | *Caragana arborescens* | ? | Missoula, MT, USA | 46.857212/ -113.988967 |
| MemA | 10 | Riverside shrubs | Ants | Austin, TX, USA | 30.267153/ -97.743057 |
| MemE | 12 | Roadside shrubs | Ants | Near Pontiac, IL, USA | 40.828982/ -88.686859 |
| MemM | 16 | Agricultural field | none | Williams, CA, USA | 39.154129/-122.150879 |
| Pub | 3 | *Lespedeza* striata | Ants | Hartwell, GA, USA | 34.376365/ -82.910727 |

**Supplementary Table 2**: Sequence and assembly data, including raw reads per sample and assembly statistics (for scaffolds > 500 bp), and coverage of cytochrome oxidase I (COI) from membracids and *Sulcia* symbionts, with NCBI SRA accessions.

| **Sample Name** | **Total**  **Reads**  **(millions)** | **# Scaffolds** | **Total**  **Assembly**  **Length** | **Maximum Scaffold Length** | **Insect COI Coverage** | ***Sulcia* Coverage** | **NCBI Accession** |
| --- | --- | --- | --- | --- | --- | --- | --- |
| BM11 | 79.23 | 257988 | 177064208 | 642820 | 616.9 | 156.6 | SAMN19458266 |
| BM13-1 | 86.30 | 647634 | 541196519 | 204188 | 1099.8 | 129.9 | SAMN19458267 |
| BM13-2 | 47.40 | 239625 | 171297124 | 77166 | 237.0 | 43.4 | SAMN19458268 |
| BM4 | 77.56 | 303426 | 230349161 | 466471 | 145.5 | 48.6 | SAMN19458269 |
| BM43 | 74.50 | 254828 | 183257314 | 327915 | 1076.7 | 178.3 | SAMN19458270 |
| BM44 | 47.94 | 166299 | 121924080 | 278817 | 600.0 | 142.0 | SAMN19458271 |
| BM50 | 67.26 | 276150 | 196178254 | 217024 | 795.4 | 298.7 | SAMN19458272 |
| BM51 | 37.34 | 124887 | 91757873 | 217024 | 443.3 | 139.4 | SAMN19458273 |
| BM53 | 67.47 | 625646 | 510935956 | 89569 | 490.9 | 165.8 | SAMN19458274 |
| BM56 | 78.58 | 729806 | 746231061 | 541920 | 912.1 | 121.4 | SAMN19458275 |
| BM59 | 105.81 | 624719 | 549064620 | 77336 | 128.1 | 77.7 | SAMN19458276 |
| BM65 | 55.45 | 617216 | 611005386 | 219575 | 651.2 | 966.3 | SAMN19458277 |
| BM69 | 74.50 | 598337 | 469213018 | 85190 | 555.6 | 65.6 | SAMN19458278 |
| Cer | 7.22 | 8111 | 7501355 | 12186 | 147.0 | 100.5 | SAMN19458279 |
| Ent | 3.77 | 7284 | 7158202 | 19329 | 114.2 | 18.5 | SAMN19458280 |
| Gar | 1.55 | 6015 | 6370930 | 27216 | 56.3 | 5.8 | SAMN19458281 |
| MemA | 111.70 | 499859 | 623057670 | 216537 | 1445.5 | 793.9 | SAMN19458282 |
| MemE | 142.01 | 533238 | 832616434 | 219407 | 7887.6 | 887.1 | SAMN19458283 |
| MemM | 486.95 | 2085838 | 2628999874 | 216046 | 2531.5 | 1270.4 | SAMN19458284 |
| Pub | 1.96 | 3059 | 3262309 | 16142 | 37.7 | 39.7 | SAMN19458285 |

**Supplementary Figure 1:** Phylogeny of *Sulcia* based on 1,426 aligned positions of the 16S rRNA gene from membracid samples in this study compared with sequences from GenBank. Maximum likelihood phylogeny reconstruction was performed in RAxML GTR+Gamma with 100 bootstrap replicates (shown on branches); with most supported nodes consistent with those obtained for the same alignment analyzed using Bayesian 50% majority rule in MrBayes with GTR+G with 4 rate categories model. Sample names and host taxon names for sequences obtained in this study are indicated in bold blue font.

**
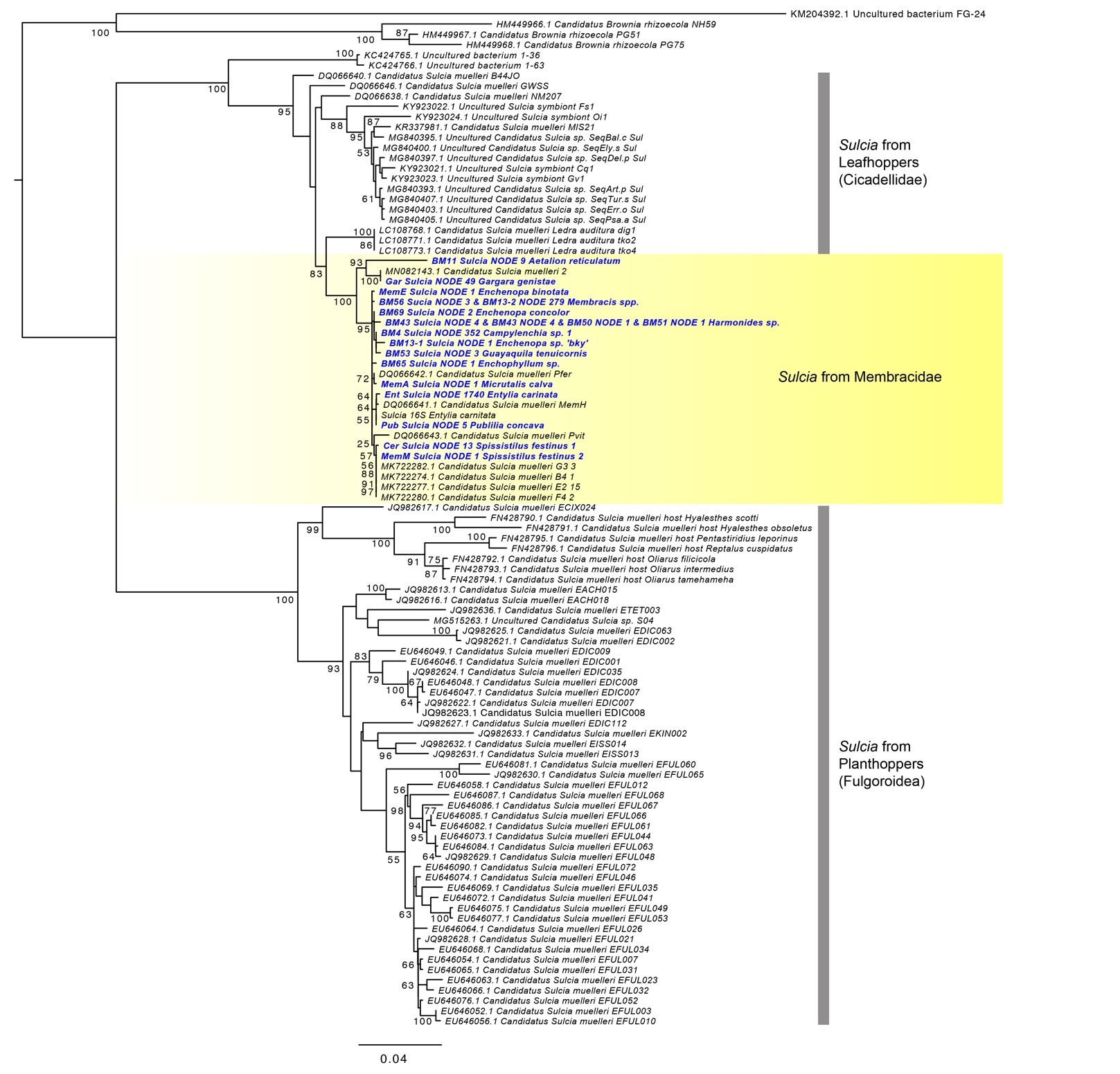
**

**Supplementary Figure 2:** Phylogeny of *Nasuia* and other betaproteobacteria based on 1,771 aligned positions of the 16S rRNA gene from membracid samples in this study compared with sequences from GenBank. Maximum likelihood phylogeny reconstruction was performed in RAxML GTR+Gamma with 100 bootstrap replicates (shown on branches); with most supported nodes consistent with those obtained for the same alignment analyzed using Bayesian 50% majority rule in MrBayes with GTR+G with 4 rate categories model. Sample names and host taxon names for sequences obtained in this study are indicated in bold blue font.


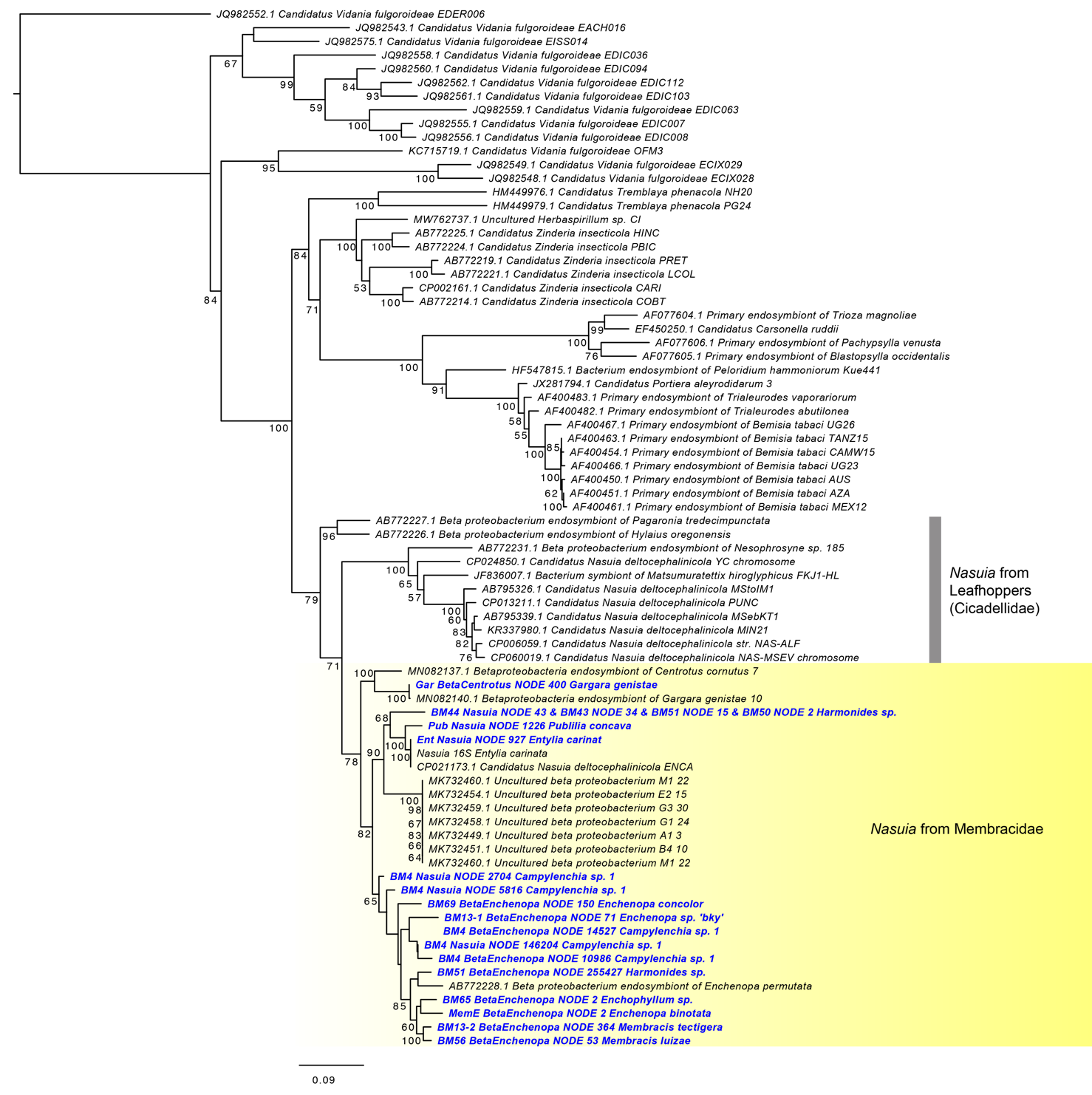


**Supplementary Figure 3:** Phylogeny of *Arsenophonus*-like sequences, including partial sequences, based on 1,494 aligned positions of the 16S rRNA gene from membracid samples in this study compared with sequences from GenBank. Maximum likelihood phylogeny reconstruction was performed in RAxML GTR+Gamma with 100 bootstrap replicates (shown on branches); with most supported nodes consistent with those obtained for the same alignment analyzed using Bayesian 50% majority rule in MrBayes with GTR+G with 4 rate categories model. Sample names and host taxon names for sequences obtained in this study are indicated in bold blue font.

**
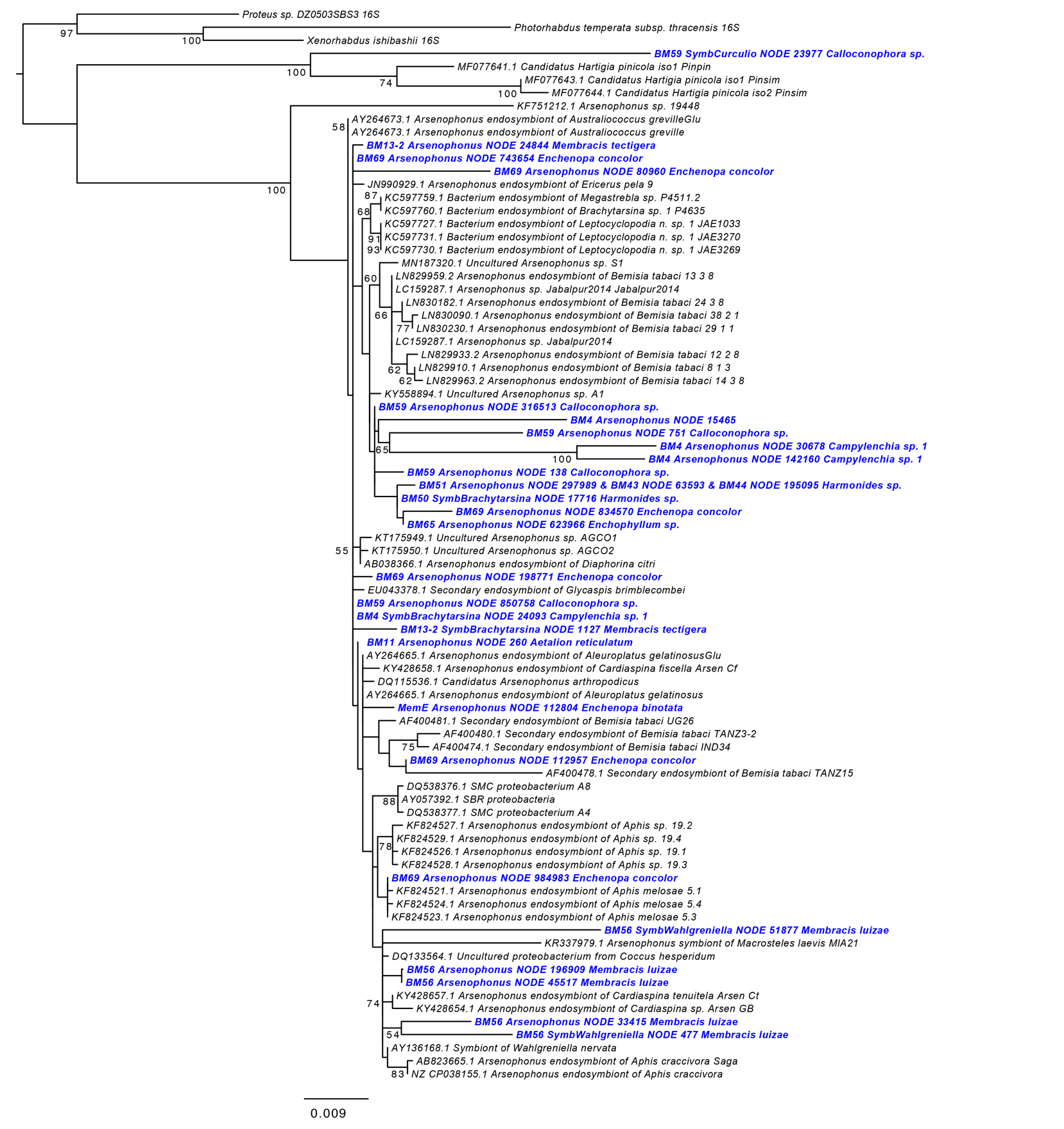
**

**Supplementary Figure 4:** Phylogeny of *Sodalis*-like sequences based on 1,579 aligned positions of the 16S rRNA gene from membracid samples in this study compared with sequences from GenBank. Maximum likelihood phylogeny reconstruction was performed in RAxML GTR+Gamma with 100 bootstrap replicates (shown on branches); with most supported nodes consistent with those obtained for the same alignment analyzed using Bayesian 50% majority rule in MrBayes with GTR+G with 4 rate categories model. Sample names and host taxon names for sequences obtained in this study are indicated in bold blue font.

**
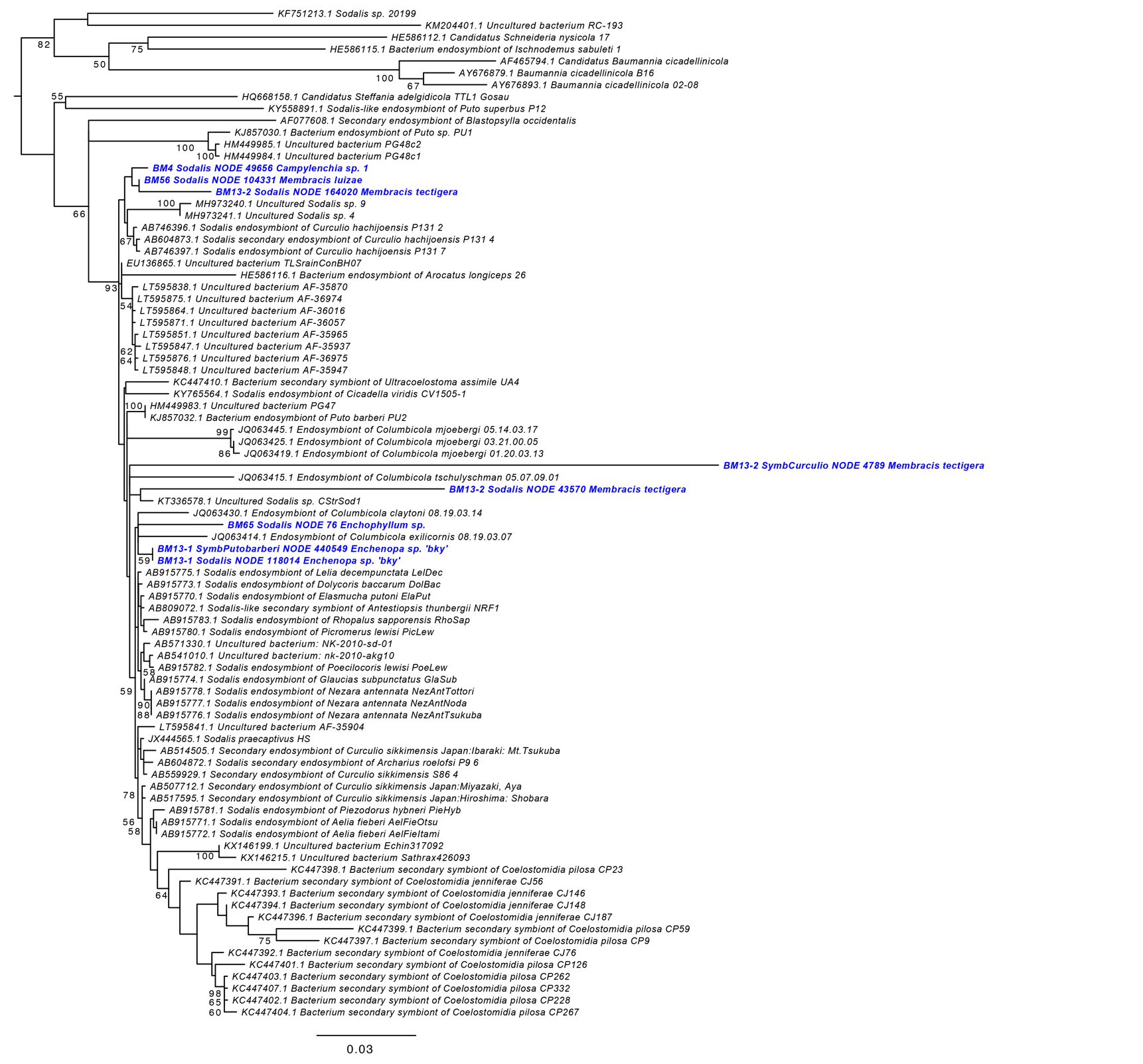
**

**Supplementary Figure 5:** Phylogeny of *Rickettsia*-like sequences based on 1,364 aligned positions of the 16S rRNA gene from membracid samples in this study compared with sequences from GenBank. Maximum likelihood phylogeny reconstruction was performed in RAxML GTR+Gamma with 100 bootstrap replicates (shown on branches); with most supported nodes consistent with those obtained for the same alignment analyzed using Bayesian 50% majority rule in MrBayes with GTR+G with 4 rate categories model. Sample names and host taxon names for sequences obtained in this study are indicated in bold blue font.

**
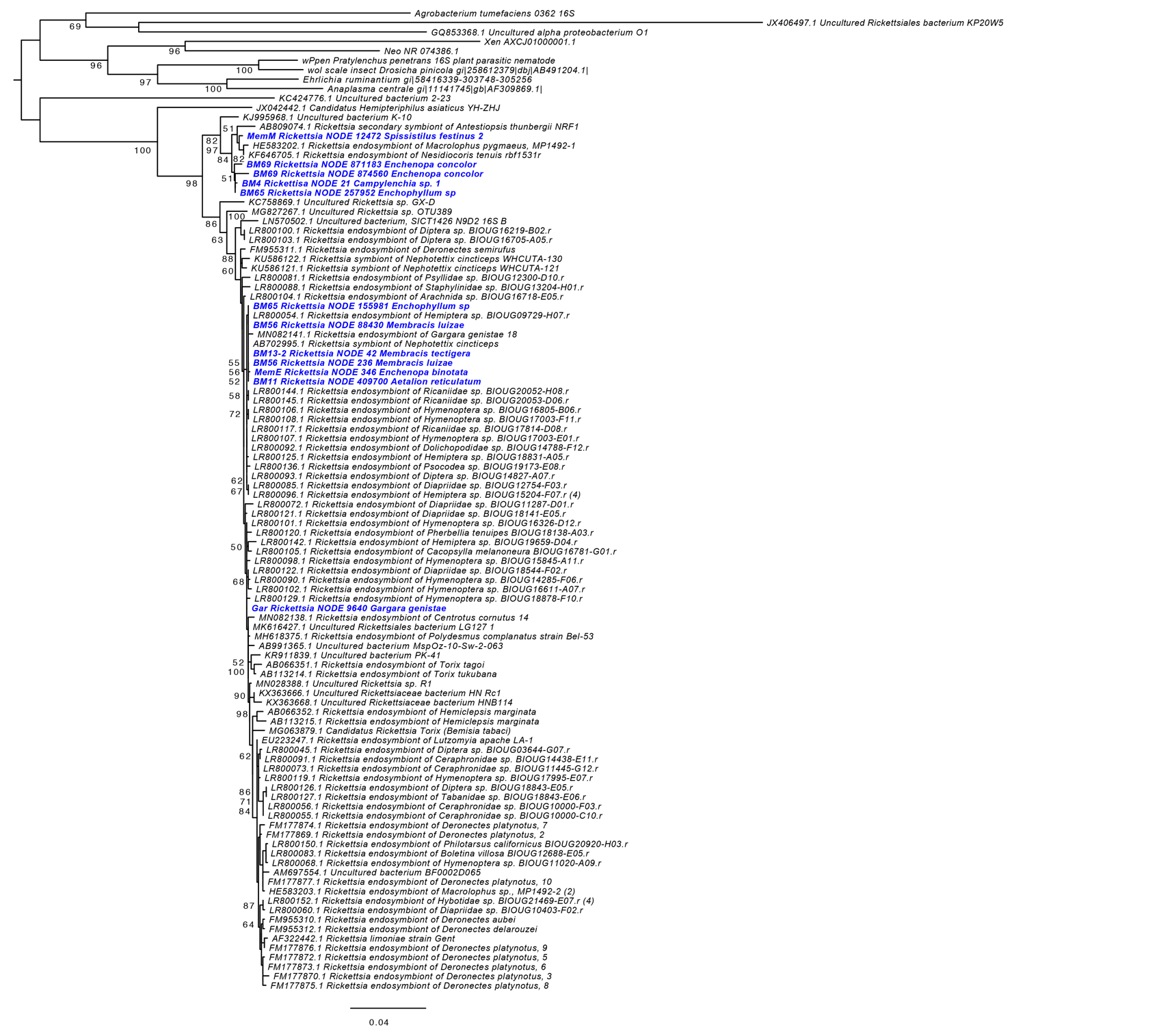
**

**Supplementary Figure 6:** Phylogeny of *Wolbachia* based on 1,305 aligned positions of the 16S rRNA gene from membracid samples in this study compared with sequences from GenBank. Maximum likelihood phylogeny reconstruction was performed in RAxML GTR+Gamma with 100 bootstrap replicates (shown on branches); with most supported nodes consistent with those obtained for the same alignment analyzed using Bayesian 50% majority rule in MrBayes with GTR+G with 4 rate categories model. Sample names and host taxon names for sequences obtained in this study are indicated in bold blue font.

**
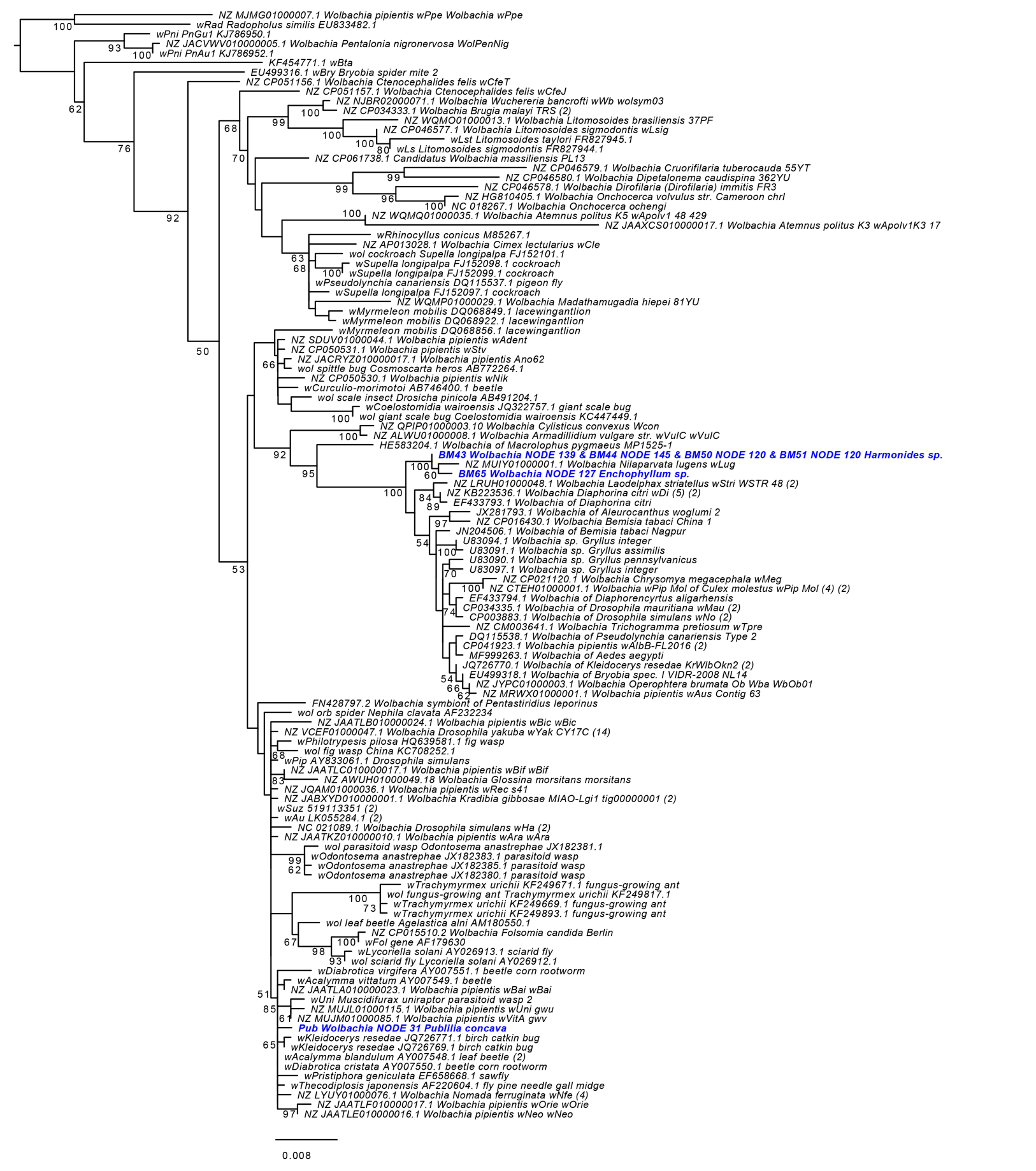
**

**Supplementary Figure 7:** Phylogeny of *Bombella*-like and *Asaia*-like sequences and related Acetobacteraceae based on 1,512 aligned positions of the 16S rRNA gene from membracid samples in this study compared with sequences from GenBank. Maximum likelihood phylogeny reconstruction was performed in RAxML GTR+Gamma with 100 bootstrap replicates (shown on branches); with most supported nodes consistent with those obtained for the same alignment analyzed using Bayesian 50% majority rule in MrBayes with GTR+G with 4 rate categories model. Sample names and host taxon names for sequences obtained in this study are indicated in bold blue font.

**
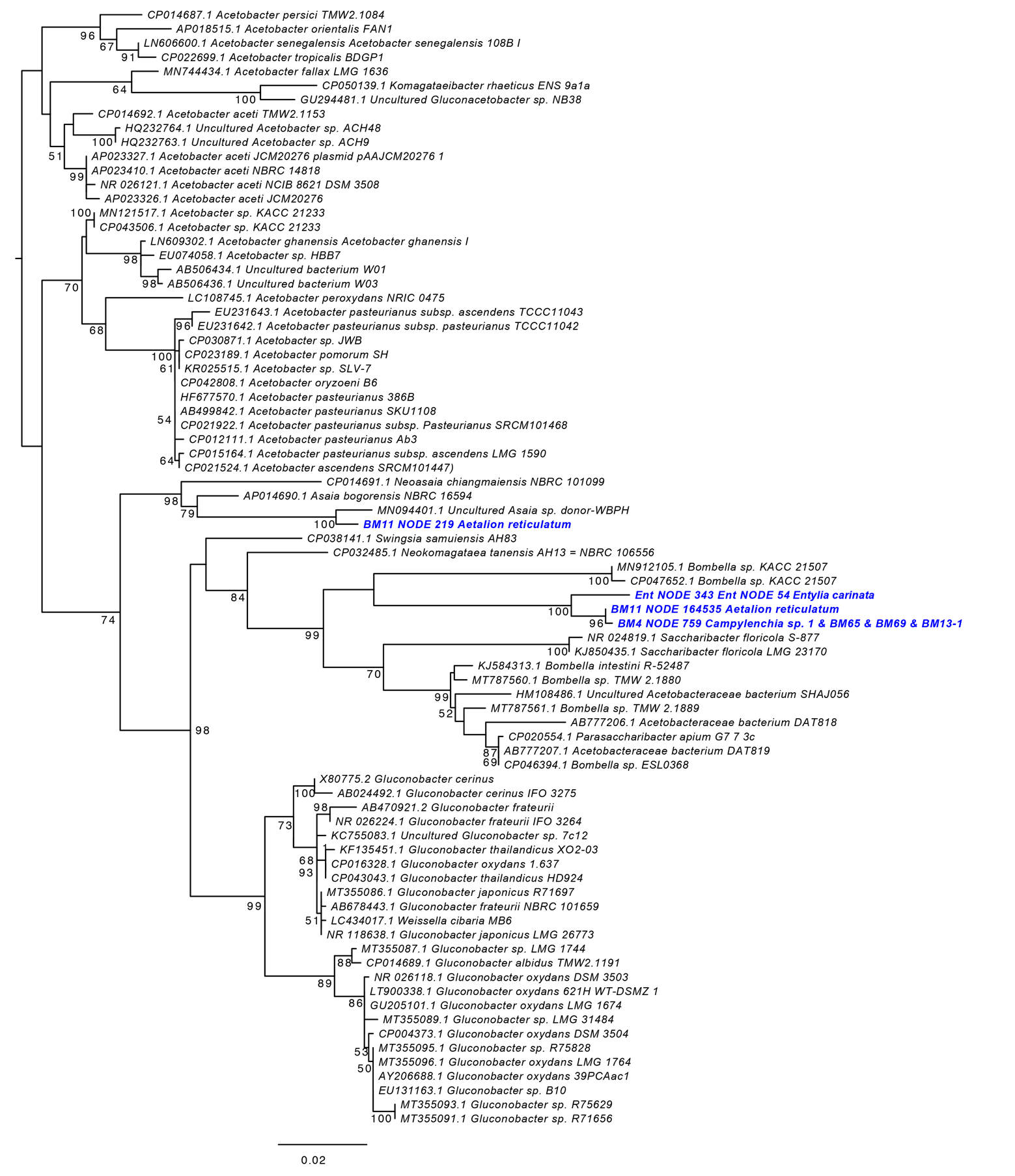
**

**Supplementary Figure 8:** Phylogeny of *Ophiocordyceps*-like fungi and yeast-like symbionts based on 4,262 aligned positions of the 18S rRNA gene from membracid samples in this study compared with sequences from GenBank. Maximum likelihood phylogeny reconstruction was performed in RAxML GTR+Gamma with 100 bootstrap replicates (shown on branches); with most supported nodes consistent with those obtained for the same alignment analyzed using Bayesian 50% majority rule in MrBayes with GTR+G with 4 rate categories model. Sample names and host taxon names for sequences obtained in this study are indicated in bold blue font.

**
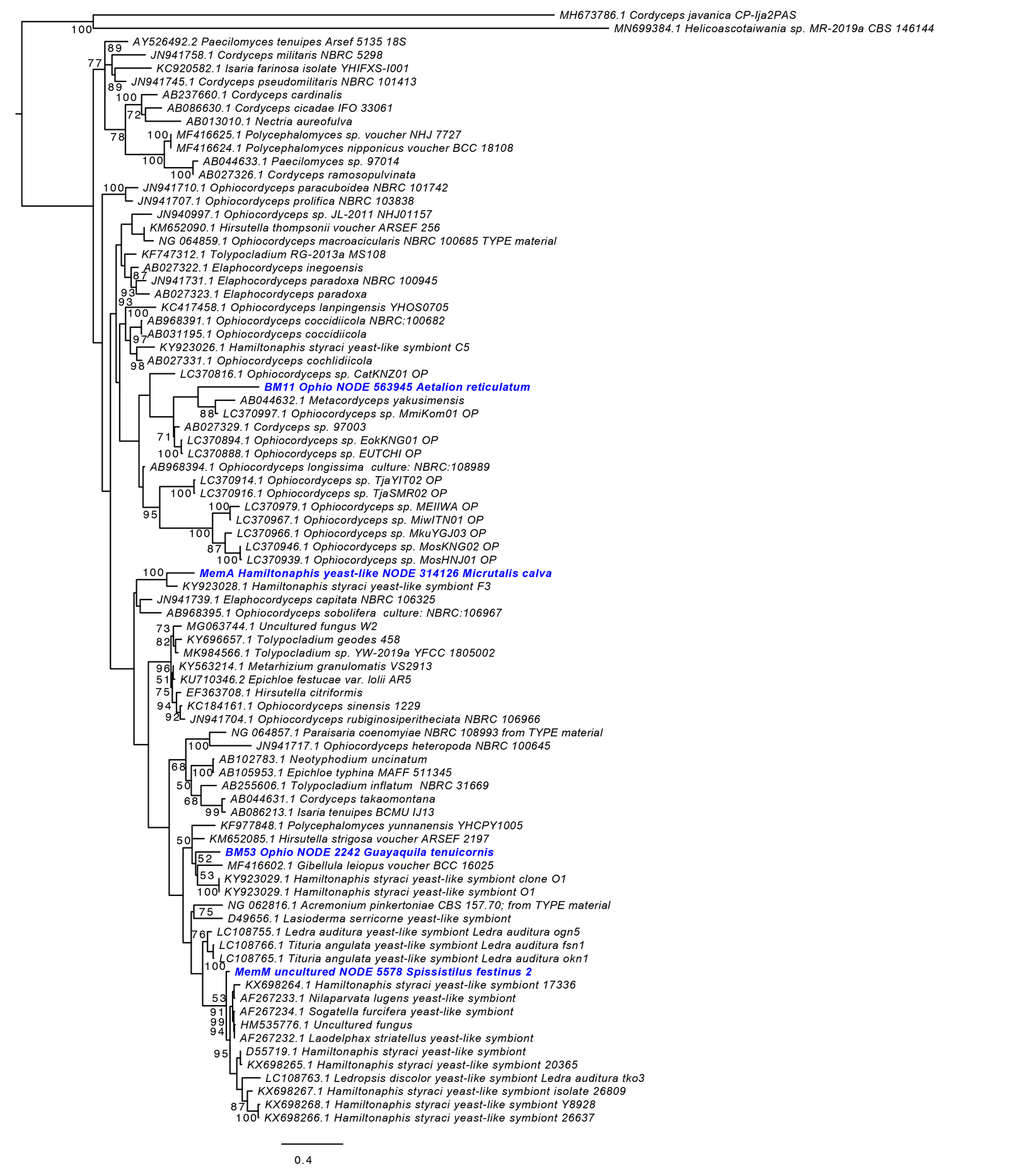
**

**Supplementary Figure 9:** Phylogeny of *Ophiocordyceps*-like fungi and yeast-like symbionts based on 1,345 aligned positions of the 28S rRNA gene from membracid samples in this study compared with sequences from GenBank. Maximum likelihood phylogeny reconstruction was performed in RAxML GTR+Gamma with 100 bootstrap replicates (shown on branches); with most supported nodes consistent with those obtained for the same alignment analyzed using Bayesian 50% majority rule in MrBayes with GTR+G with 4 rate categories model. Sample names and host taxon names for sequences obtained in this study are indicated in bold blue font.


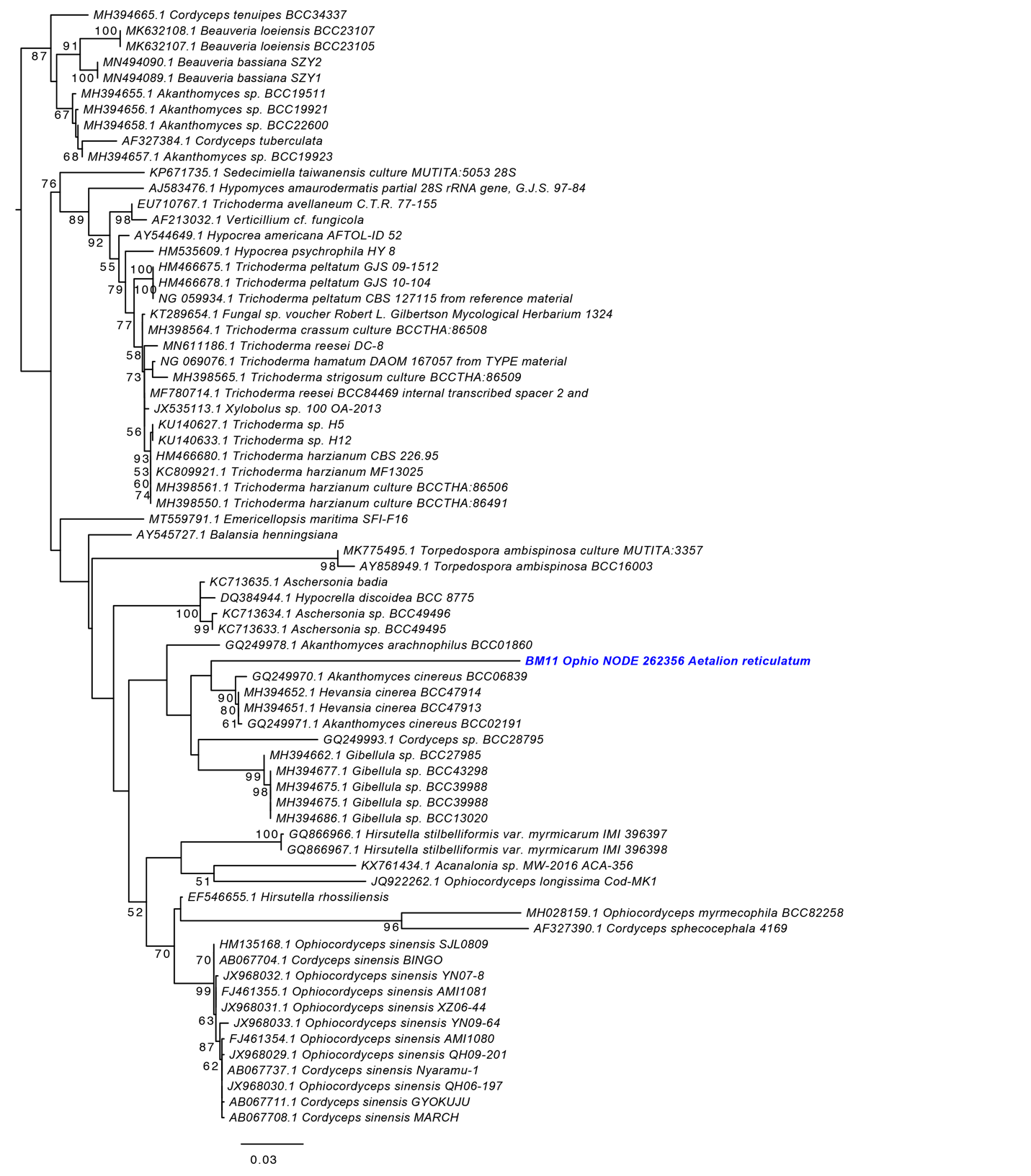

Supplement: Supplementary file 1 [file Table_1.docx]
